# Countrywide *Corchorus olitorius* L. core collection shows an adaptive potential for future climate in Benin

**DOI:** 10.3389/fpls.2025.1634672

**Published:** 2025-09-30

**Authors:** Dèdéou A. Tchokponhoué, Sognigbé N’Danikou, Emmanuel Omondi, Spéro Coffi, Belchrist Eliel Sossou, Aristide Carlos Houdegbe, Charlotte A. O. Adje, Nicodeme V. Fassinou Hotegni, M. Eric Schranz, Maarten van Zonneveld, Enoch G. Achigan-Dako

**Affiliations:** ^1^ Genetics, Biotechnology and Seed Science Unit, Laboratory of Plant Production, Physiology and Plant Breeding, Department of Plant Sciences, University of Abomey-Calavi, Cotonou, Benin; ^2^ World Vegetable Center, Eastern and Southern Africa, Duluti, Arusha, Tanzania; ^3^ Ecole d’Horticulture et d’Aménagement des Espaces Verts, Université Nationale d’Agriculture, Kétou, Benin; ^4^ World Vegetable Center, Headquarters, Shanhua, Tainan, Taiwan; ^5^ Biosystematics Group, Wageningen University and Research, Wageningen, Netherlands

**Keywords:** single nucleotide polymorphism, genetic diversity, core collection, germplasm conservation, genomic offset analysis, jute mallow

## Abstract

**Introduction:**

Understanding the genome-wide variation pattern in crop germplasm is required in profiling breeding products and defining conservation units. Yet, such knowledge was missing for the large germplasm collection of *Corchorus olitorius* in Benin at CalaviGen (the University of Abomey-Calavi genebank), the world’s largest holder of the crop germplasm with 1,566 accessions conserved.

**Methods:**

Using 1,114 high-quality SNPs, this study: i) investigated the spatial variation of the genetic structure of 305 accessions sampled along the South-North ecological gradient of Benin, ii) derived a core collection from the batch of accessions and iii) gauged the extent of (mal)adaptation of this core set.

**Results and discussion:**

Overall, we detected a moderate diversity with a total gene diversity of 0.28 and an expected heterozygosity estimate of 0.27. The spatial variation of the genomic diversity painted an increasing trend following the South-North ecological gradient, giving rise to four optimal genetic groups based on STRUCTURE analysis while the neighbour-joining analysis revealed three clusters. The ShinyCore algorithm application yielded a core set of 54 accessions that echoed a good geographical representativeness and encompassed a level of diversity comparable to that of the whole collection. Nearly 88% of this core set accessions were characterized by a low genomic offset score, which suggests a strong adaptation potential to future climate. This SNP-based core collection represents a unique and viable working asset for accelerated traits-discovery, in the species and should play a pivotal role in international collaborative initiatives dedicated to promoting *C. olitorius* use and conservation.

## 1 Introduction

A core collection (CC) in plant genetic resources management is a representative set with the maximum possible diversity and the lowest possible redundancy extracted from a whole and larger germplasm. It is a concept introduced by Frankel (1984) to rationalize germplasm management. In genebank, core collections are used to optimize genetic resources management under constraints and to sustain conservation efforts. Examples of well-known genebank core collections included the AfricaRice *Oryza sativa* L. core collection (Ndjiondjop et al., 2023), the International Institute for Tropical Agriculture (IITA) cowpea [*Vigna unguiculata* (L.) Walp.] core collection (Mahalakshmi et al., 2007), the United States National Plant Germplasm System (NPGS) cucumber (*Cucumis sativus* L.) core collection (Wang et al., 2018), among others.

For breeders and researchers, core collections represent fine-tuned working sets for accelerated trait discovery and many other applications (Gu et al., 2023). For instance, in the USA, the melon (*Cucumis melo* L.) core collection was used to detect loci associated with flesh thickness, fruit flesh weight, fruit shape, fruit diameter and fruit length (Wang et al., 2017). Shi et al. (2021) and Zia et al. (2022) tapped into the United States Department of Agriculture (USDA) common bean (*Phaseolus vulgaris* L.) core collection to unravel single nucleotide polymorphism (SNP) markers associated with resistance to the soybean [*Glycine* max (L.) Merrill] cyst nematode, and bacterial wilt, respectively. Similarly, the barley (*Hordeum vulgare* L.) core collection of the same institution (USDA) has been instrumental in depicting SNPs associated with hull cover, spike row number, and heading date (Muñoz-Amatriaín et al., 2014). McLeod et al. (2023) exploited the “Linking genetic resources, genomes and phenotypes of solanaceous crops” G2P-SOL project core collection to identify over fifty genes associated with plant productivity, vigour, earliness, fruit flavour, colour, size, and shape in *Capsicum* spp. Core collection is also useful in genomic selection studies as it increases the robustness of the training population phenotyping and the identification of performing genotypes (Hong et al., 2020; Kang et al., 2023). Nowadays, the concept of “core collection” is gaining popularity in supporting global initiatives dedicated to informed conservation and use of plant genetic resources across Africa, and in which readily available germplasms encompassing a reasonable level of diversity are highly sought after. If it is true that genebanks in general hold large number of accessions for various species, core collection development remains, however, an exercise that is yet to popularize, across genebanks in Africa. Doing so will be useful for genebank operations optimization, but also will facilitate the use of genetic materials by users’ group both locally and across international initiatives.

Several methods have been historically employed to build core collections across diverse commodity groups. These were based on phenotypic data (Tchokponhoué et al., 2020) or molecular data (Schafleitner et al., 2021, 2015; Wang et al., 2023), or the integration of both (Ghamkhar et al., 2008; Lee et al., 2016). Noticeably, approaches integrating the use of molecular data, and of single nucleotide polymorphism (SNPs) data in particular, have been largely recommended (Ghamkhar et al., 2008) given the unique peculiarities of these markers to be not only salient across plant species genomes but also to offer the highest possible resolution in capturing existing diversity and population structure, a prerequisite for any core collection construction (Christov et al., 2021). In addition, unlike phenotypic information that are highly influenced by the environment, SNP are stable.

Using molecular data, and depending on the perspective, three categories of core collections can be constructed: (i) a core collection painting individual accessions in the whole core (CC-I) and which includes both common and rare alleles while preserving a minimum redundancy in accessions; (ii) a core collection representing extremes of the entire collection (CC-X) which prioritizes entries at both extreme tails of the whole collection distribution; and (iii) a core collection capturing the distribution of accessions of the whole collection (CC-D) and which maximizes the representativeness of entries with common alleles at the expense of the rare ones (Marita et al., 2000; Odong et al., 2013). The CC-I was recommended as the first order core collection category for researchers in general, and for breeders in particular (Odong et al., 2013).

The study reported in this paper used the CC-I approach to build a mini-core collection for Jute mallow (*Corchorus olitorius* L.) with the objective to make available to various end-users, especially students, breeders, and researchers fine-tuned working germplasms, but also to improve conservation options for the species at CalaviGen, the University of Abomey-Calavi genebank. CalaviGen, since 2024, stood as the worldwide leading jute mallow collection holder with 1,566 accessions from diverse phytogeographical regions of Benin (Link 1).

Conceptually, core collections come at a reduced size, and if maladapted will not sustainably serve breeding and conservation purposes. Here, we conceptualized maladaptation in the sense of Rellstab and Keller (2025) as the likelihood of disruption to adaptation arising from environmental disturbance. In a context of climate change, it then becomes imperative to gauge the extent of adaptation in core set germplasms. To the best of our knowledge, this is the first study employing an analysis that assesses a core set’s fitness for future climates. We employed a genomic offset analysis following the method of Fitzpatrick and Keller (2015).

*Corchorus olitorius* is a worldwide versatile crop valued as food, fibers, and medicinal remedies. In Africa, the leaves of the species are very appreciated for their sliminess and slenderness exploited to make sauces that accompany meals in many countries including Nigeria, Benin, Togo, Ghana and Egypt, among others (Youssef et al., 2019). It represents a good source of β−carotene, vitamins C, E, B1, B2, iron and calcium (Mahalakshmi et al., 2007). In Asia, the species produces one of the best quality vegetable fibers thanks to its lignin and cellulose contents (Kirby, 1963; Sarkar et al., 2017). *Corchorus. olitorius* is also considered as a general healer in the treatment of many diseases including constipation, heart diseases, dysentery, cystitis, diarrhea (Ahmed, 2021; Burkill, 2004) and typhoid fever (Kakpo et al., 2019). Taken together, the species leaves and flowers contained more than sixty phytochemicals conferring a wide range of pharmacological effects including hepatoprotective, antidiabetic, neuroprotective, antimicrobial (Ahmed, 2021). In Benin, *C. olitorius* is among the top four most consumed leafy vegetables in urban areas and one of the top five most widely traded vegetables (Achigan-Dako et al., 2010), and holds the potential to improve income, food security and nutrition profile in the local communities.

Knowledge of the large collection of the species currently maintained at CalaviGen is still limited; a situation that can be generalized to the Benin *Corchorus* germplasm in general. Specifically, while our knowledge of the species phenotypic diversity in Benin is still at its infancy (Adebo et al., 2015; Adjatin et al., 2019; Afokpe et al., 2025), no molecular diversity analysis has so far been reported for the species. Yet, robust information on the extent of genetic variation in the species is crucially needed to design proper breeding strategies and implement an informed conservation strategy, especially in a context of marked climate change and increasing urbanization.

*Corchorus olitorius* thrives in all three phytogeographical zones of Benin that are marked by a latitudinal ecological gradient whereby environmental conditions become drier as one moves close to the northern part. In the presence of such a gradient it is often hypothesized that there is a core-edge decline in genetic diversity (Zardi et al., 2015), but whether this trend is confirmed in the case of *C. olitorius* is yet to be demonstrated. Hence, this study seeks to: i) understand the extent to which genome-wide diversity in *C. olitorius* evolved along the south-north ecological gradient observed in Benin; ii) reveal how the South-North gradient is reflected in the species population structuring pattern, iii) highlight how much variation of the studied collection is captured in the core set, and iv) understand how the derived core set population can adapt to environmental conditions changes.

## 2 Materials and methods

### 2.1 Plant material and sampling sites

**Figure 1:**
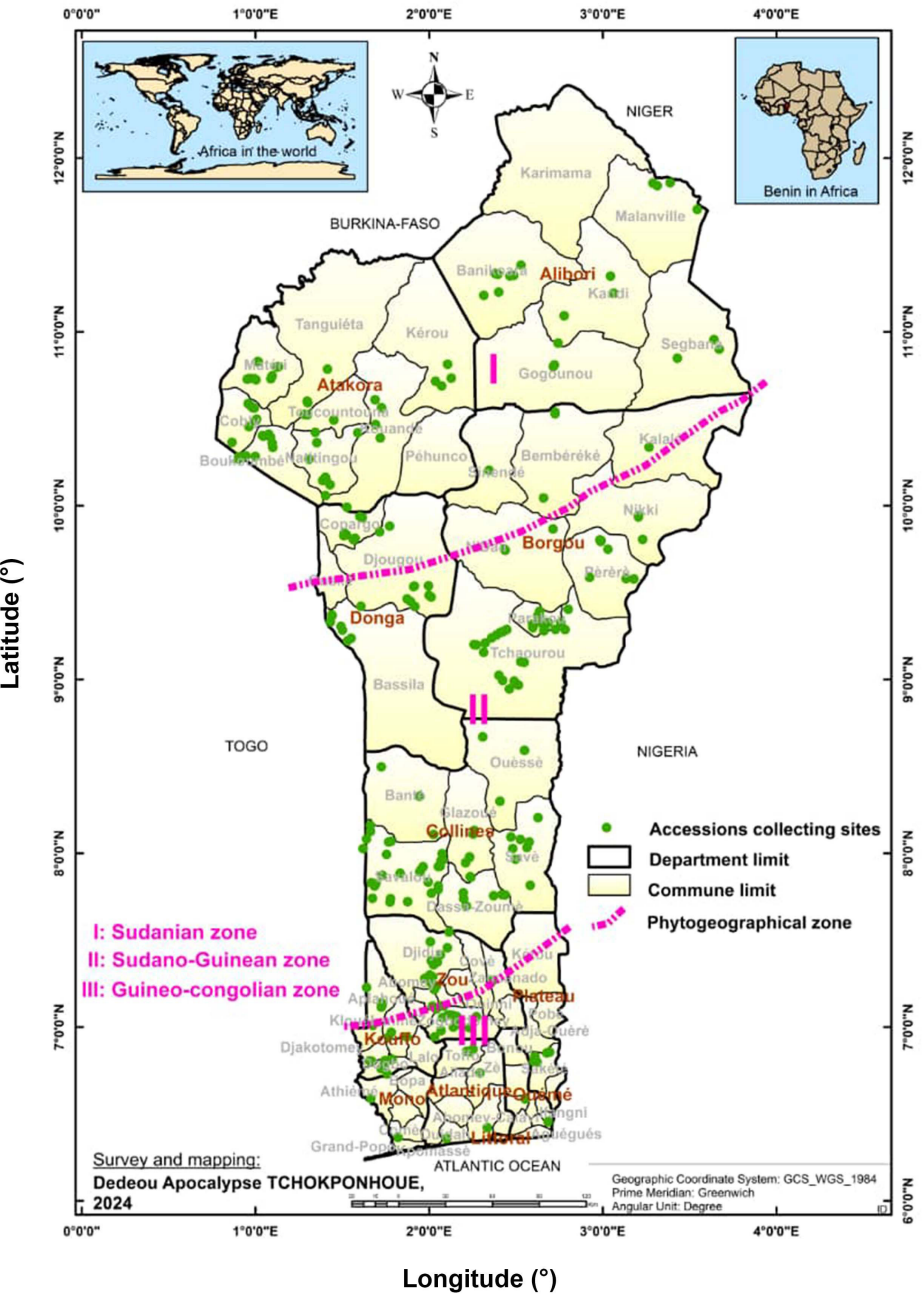
Map showing the distribution of the collecting points across the three geographical populations of *Corchorus olitorius* in Benin. The seed samples collection was carried out from 2021 to 2023.

The plant material used consisted of a total of 305 accessions sampled from the CalaviGen collection of *C. olitorius* (of a total size of 1,566 accessions) to represent the three phytogeographical zones of Benin, namely the Guineo-Conglian (GC), the Sudano-Guinean (SG) and the Sudanian (Su) zones. These accessions were collected from various habitats including farmers’ fields and home gardens during a country-wide prospecting and collecting missions organized from 2021 to 2023. The three phytogeographical zones also stood here as three distinct geographical populations. Here, a population was defined in the sense of Tchokponhoué et al. (2023) as a group of accessions distributed within a specific phytogeographical zone and possibly intermating. Out of these 305 accessions, 51 were collected from the GC population, 152 from the SG and 102 from the Su population (**Supplementary Table S1**). These sample sizes were proportional to the total number of accessions held from each phytogeographical region by CalaviGen. The distribution map of the accession collecting sites is presented in **Figure 1**. During the collecting, we kept a minimum distance of 200 meters between two collecting sites to avoid sampling related accessions while a minimum distance of 15 km separated accessions from two different populations.

### 2.2 Tissue sampling and genotyping

The 305 accessions were raised for two weeks in a nursery for leaf tissue sampling. Three to five newly emerged and pest-free leaves were sampled for each accession, placed into light envelopes, and automatically deposited on silica-gel for DNA preservation. The leaves batches gathered were labelled and shipped to SEQART AFRICA [the African branch of the Diversity Arrays Technology Pty. Ltd. Sequencing Company based in Canberra, Australia] for further processing and sequencing based on the Diversity Array Technology Sequencing (DArTseq) platform. This additional processing consisted in the subsampling of the leaves into four 96 well plates, each receiving 94 accessions, with the last two wells reserved for controls. The plates were then submitted to automatized DNA extraction as per the NucleoMag^®^ Plant procedure (**Supplementary Table S2**). The DNA was restricted using enzymes Pst1 and Mse1, followed by a genotyping based on the DArTseq™ pipeline. A high-density sequencing targeting 2,500,000 reads per sample was carried out on the Illumina Hiseq 250 platform. Finally, raw reads were processed. SNP markers were called using the in-house Diversity Array Technology’s proprietary analytical pipelines (Sansaloni et al., 2011). The markers were mapped to the chromosome-level reference genome of *C. olitorius* (Zhang et al., 2021) (Link 2).

### 2.3 Bioinformatics

The raw sequencing report consisted of a single nucleotide polymorphism matrix where each accession for each SNP was scored as either 0 (reference homozygous allele), 1 (alternative homozygous allele), 2 (heterozygous allele), or NA (for missing data) together with the SNP metadata. This genomic matrix was first filtered using the R “dartR.base” package (Gruber et al., 2023). We filtered the genomic matrix for SNPs with i) a minor allele frequency (MAF) < 0.01, ii) a call rate < 75% and iii) a reproducibility rate < 80%. Then, we imputed the missing data using the Singular Value Decomposition (SVD) algorithm on the KDcompute platform (https://kdcompute.seqart.net/kdcompute/login) and recomputed the SNP metadata using the *recal.metrics ()* function of the same “dartR.base” package. The full genomic matrix thus obtained was then considered for the downstream analyses.

#### 2.3.1 Population diversity

Overall genomic diversity statistics including observed heterozygosity (Ho), expected heterozygosity (Hs), total gene diversity (Ht) and inbreeding coefficient (Fis) were computed using the function *get.snpR.stats ()* in the “snpR” package (Hemstrom and Jones, 2023) to understand the overall diversity in *C. olitorius* germplasm. The variation across the geographical populations of the diversity statistics Ho, Hs and Fis were computed using the function *gl.report.heterozygosity ()* of the R “dartR.base” package and then standardized where necessary (Ho and Hs) by integrating the number of invariant loci obtained from the function *gl.report.secondaries ()* of the same package. The significance of these statistics variation among populations was tested with the function *gl.test.heterozygosity ()* still in “dartR.base” using 1,000 replications of re-randomization.

#### 2.3.2 Population differentiation and structure

We assessed the overall population differentiation (Fstg) using the function *get.snpR.stats ()* in the “snpR” package. To compute and plot the pairwise population differentiation index (Fstp) across pairs of populations, we applied the functions *calculate_pairwise_fst ()* and *plot_pairwise_fst_heatmap ()* of the “snpR” package, respectively.

Population structure was assessed using a combined approach of STRUCTURE analysis (Pritchard et al., 2000) and analysis of molecular variance (AMOVA). We performed the STRUCURE analysis run with a 10,000 Markov chain Monte Carlo length and a 10,000 iterations burn-in-period on three independent replicates of K values, with K = 1 - 10. The results of the analysis were used to determine the optimal number of clusters following the delta K method (Evanno et al., 2005) implemented in the “Pophelper” package (Francis, 2017) through the *evannoMethodStructure ()* function. The same package was also used to plot the accessions assignment to various genetic groups based of the optimal number of clusters detected. An accession was assigned to a specific group if only its membership coefficient for this group was ≥ 0.6 (Coulon et al., 2008; Girma et al., 2024). A fisher.exact test was then used to test the genetic membership’s association with the geographical origin. For the AMOVA, a hierarchical model was employed through the *poppr.amova ()* function of the “Poppr” package (Kamvar et al., 2024) to partition the genetic variation across geographical populations and individuals while the significance of each source of variation was tested using the *rand.test ()* function with 1,000 bootstrap replications.

#### 2.3.3 Phylogenetic relationships among accessions

The relationship among accessions was depicted using a neighbour-joining analysis (Saitoh and Nei, 1987) based on the Tajima-Nei model (Tajima and Nei, 1984) implemented in the Molecular Evolutionary Genetic Analysis (MEGA V.11) software (Tamura et al., 2021). The robustness of the phylogeny model was gauged using 1,000 bootstrap replications. The tree Newick file was further processed on the Interactive Tree of Life (V6) (Letunic and Bork, 2024) platform.

#### 2.3.4 Core set development and evaluation

The core set was developed from the collection of 305 accessions using the recently published “ShinyCore” R package (Kim et al., 2023), a program specifically designed to build core collections based on SNP data. The ShinyCore algorithm implemented a two-phase brute-force approach. The first phase optimizes allele coverage (the proportion of marker alleles in the entire collection that are retained in the core collection). It maximizes allele rarity in its second phase. Contrary to existing algorithms such as Core Hunter (De Beukelaer et al., 2018), and GenoCore (Jeong et al., 2017), ShinyCore offers the flexibility to pre-define a maximum (z) and a minimum (y) coverage threshold, to extract the final core set by topping-up a pre-final core set of size Ny (obtained at y% coverage threshold) with the (Nz-Ny) accessions with the highest total rarity score. This study sets y and z at 98 and 99, respectively. ShinyCore was proved to outperform the well-known GenoCore algorithm in core set development (Kim et al., 2023).

The core set was evaluated by comparing overall genetic statistics (Hs, Ht) between the whole and the built core collections.

#### 2.3.5 Genotype-environment association and outlier analysis of core set

We integrated the genotype-environment association analysis and outlier methods (PCAdapt) to identify the signatures of selection and environmental adaptation in our core set following Omondi et al. (2025). This combined analysis approach allows to maximally capture the signals of selection due to the different working backgrounds of the methods. The genotype-environment association analysis combined the genomic matrix and the environmental variables of the georeferenced core set individuals to detect adaptive signals from SNPs of which the allele frequency is associated with environmental variables (Rellstab et al., 2015). PCAdapt on the other hand is an outlier analysis that scans for loci that deviate from the expected pattern of neutral genetic variation, which could indicate adaptation that is correlated to a particular population structure.

##### 2.3.5.1 Environmental variables selection

The geographical coordinates including latitude and longitude of the retained individuals were used to retrieve the environmental data from the WorldClim 2.1 (Fick and Hijmans, 2017) at a resolution of 2.5 min and individually averaged over the period between 1970 and 2000 for the historical (near current) climate data. Additionally, the future climate predictions were downloaded. For this, we used the statistically downscaled, bias-corrected CMIP6 global climate models (GCMs) at 2.5 min resolution. We averaged three of the GCMs (BCC-CSM2-MR, MRI-ESM2–0 and IPSL-CM6A-LR) for the time period between 2061–2080 and for two shared socioeconomic pathways (SSPs) - SSP370 and SSP585 to account for uncertainty in the model projections of the future climate. Finally, soil data were downloaded from the SoilGrids database (https://soilgrids.org/) (Hengl et al., 2017) at a 250-meter resolution. All the climate and soil rasters were combined after bringing them to the same resolution and projection using the “terra” R package (Hijmans et al., 2023). After retrieving the environmental variables values using the coordinates of the core set entries, the environmental variables were further pruned considering a variance inflation factor of 2. All the environmental variables were scaled and centred for downstream analysis.

##### 2.3.5.2 Genotype environment association analysis and outlier analysis

We applied two Environment Association analysis (EAA methods - redundancy analysis (RDA) (Legendre and Legendre, 2012) and latent factor mixed model (LFMM) (Caye et al., 2019) − to estimate the amount of genomic variation attributable to the environment (climate and soil), geography and genetic structure. The two methods are in principle similar except that while RDA assesses linear relationship between two or more variables at the same time, LFMM examines the linear relationship between two variables at a time. Both methods allow for controlling the population structure to reduce false positives rates when detecting the signals of adaptation. In both methods, the SNP matrix was used as the response variable while the environmental data variables (historical climate and soil data) as the explanatory variables. In a partial RDA, variance partitioning was done by applying the *rda ()* function on the “vegan” R package (Oksanen et al., 2007). We further tested the significance of the partial RDA models using the R function *anova.cca ()* with 5,000 permutations. The candidate adaptive SNPs in the partial RDA model were identified as those having a loading of ± 2 standard deviations of the first three significant axes using the *outlier ()* function following Capblancq et al. (2018).

The LFMM was performed using the “LFFM” R package (Caye et al., 2019). We used the optimal K initially determined in the population structure analysis to control for population structure. We considered SNPs to be candidate adaptive SNPs at a false discovery rate (FDR) < 0.05.

For the outlier analysis method, we applied the *PCAdapt ()* function using the “PCAdapt” R package (Luu et al., 2017). This method simultaneously uses a PCA-based approach to infer a population structure and identify the outlier SNPs correlating to the structure. To detect the outliers, we adjusted the p-values using the Bonferroni method in the *p.adjust ()* function of the “qvalue” R package (Storey et al., 2015) and then applied the FDR threshold of ≤ 0.05.

Finally, we combined the results of the partial RDA, LFMM and PCAdapt and used this set of candidate adaptive SNPs for the subsequent analyses.

##### 2.3.5.3 Genomic offset under future climates

For this analysis, our interest was to show the core set entries that might be at high risk of future maladaptation and the spatial regions of the landscape that are highly likely to be affected by future climates following the idea of Rellstab et al. (2016). To derive the adaptive landscape, we solely focused on the outlier SNPs identified by the EAA and Outlier Analysis (OA) methods. A subset of the outlier overlapping among the three methods would have been the best to focus on but we detected very few (just 4 SNPs). With the new genomic data set of the outlier SNPs, we ran the “adaptively enriched” RDA this time without conditioning for population structure. Thereafter we applied the *genomic_offset ()* function (Capblancq and Forester, 2021) to predict the genomic offset based on a RDA model. This function returns projections for current and future climates for each RDA axis, prediction of genetic offset for each RDA axis and a global genetic offset (combined prediction for all the significant axes). In our case we selected the first two significant axes to build our global prediction. To visualize the genomic vulnerability on the landscape, we plotted a raster image of the projection of the global genomic offset for the future environmental conditions using the *ggplot ()* function of the “ggplot2” R package (Wickham et al., 2016) after which we superimposed the collection sites of the core set entries. The color bands for the plot were rescaled to 0.5 value ranges prior to the plotting. High values of the genomic offset indicate a low adaptation capacity to the future climates.

## 3 Results

### 3.1 Markers quality

**Figure 2:**
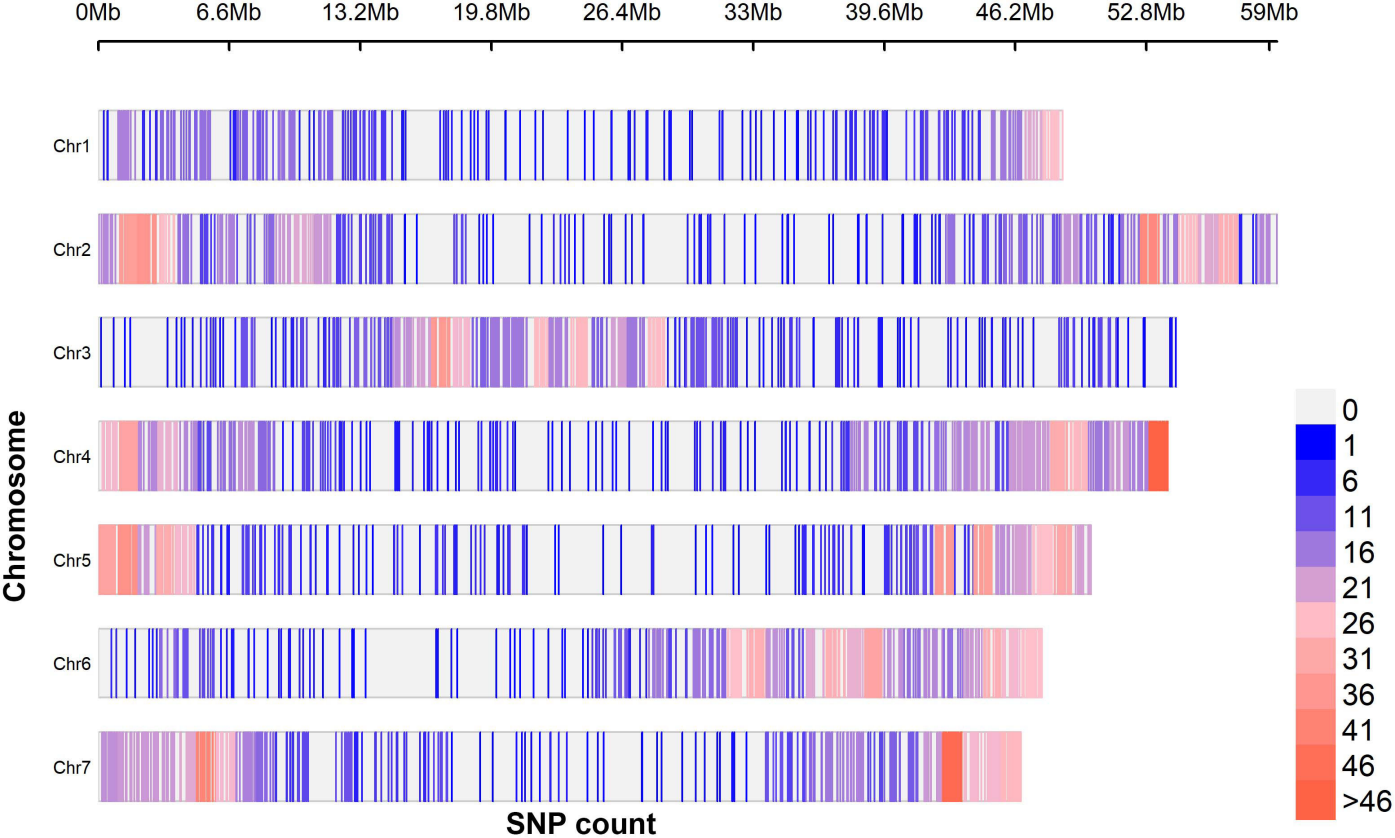
Single nucleotide polymorphism markers density variation along *C. olitorius* chromosomes using 1MB sliding window. A total of 3,575 SNPs were generated from a batch of 305 accessions of *C. olitorius*.

A total of 4,340 raw single nucleotide polymorphism (SNP) (**Supplementary Table S3**) markers were generated from the sequencing of which 3,575 (82%) were effectively aligned to the *C.olitorius* genome. The distribution of these SNPs along the species chromosomes is presented in **Figure 2**. Markers density ranged from SNP/140.55 kbp (chromosome 1) to SNP/87.05 kbp (chromosome 7), suggesting chromosome 7 as the densest. The filtering process resulted in a narrowed-down number of 1,114 SNPs that were used in the subsequent analyses. These markers minor allele frequency (MAF) values ranged from 0.01 to 0.5 for an average of 0.20 ± 0.04 while their polymorphism information content (PIC) values were between 0.02 to 0.49 for an average value of 0.27 ± 0.05 (**Supplementary Figure S1**). The percentage transition mutation (Ts) and transversion mutation (Tv) among the markers were 57.18% and 42.82%, respectively, giving rise a Ts/Tv ratio of 1.34.

### 3.2 Population diversity and differentiation

Based on the retained markers, values of observed heterozygosity (Ho), expected heterozygosity (Hs), and total gene diversity (Ht) in *C. olitorius* were 0.07, 0.27 and 0.28, respectively. The inbreeding coefficient was estimated at 0.72. The disaggregation of heterozygosity and inbreeding coefficient estimates across the three geographical populations (**Table 1**) suggested a significant south-north trend in diversity increase (p = 0.02) with the Sudanian region’s accessions exhibiting the highest expected heterozygosity. In contrast, the Guineo-Congolian accessions presented the lowest one. This trend of increasing diversity from the southern to the northern part of the country was maintained even with the autosomal heterozygosity (**Table 1**). Nevertheless, the allelic richness (Ar) was similar among the three populations (p = 0.05).

**Table 1:** Population diversity statistics of the 305 accessions of jute mallow (*Corchorus olitorius*) based on 1,114 single nucleotide polymorphism markers.

Population	Ho	Hs	Fis	Ar	HsAdj
Guineo-Congolian (GC)	0.087 ± 0.002	0.266^b^ ± 0.005	0.67 ± 0.006	1.92	0.00327 ± 0.001
Sudano-Guinean (SG)	0.077 ± 0.001	0.268^ab^ ± 0.005	0.71 ± 0.006	1.95	0.00333 ± 0.001
Sudanian (Su)	0.063 ± 0.001	0.274^a^ ± 0.005	0.76 ± 0.005	1.94	0.00338 ± 0.001

Ho, Observed heterozygosity; Hs, Expected heterozygosity; Fis, Inbreeding coefficient; Ar, Allelic richness; and HsAdj, Autosomal heterozygosity. Values with the same superscript letters in the same column are not statistically different at 5%. Dispersion measure is standard error.

*Corchorus olitorius* was a low-differentiated species as suggested by the overall Fstg value of 0.04. Pairwise-taken, the Guineo-Congolian and the Sudanian populations were the most differentiated populations whereas the Sudano-Guinean and the Guineo-Congolian ones stood as the least differentiated ones (**Supplementary Figure S2**).

### 3.3 Population structure and phylogenetic relationship

**Figure 3:**
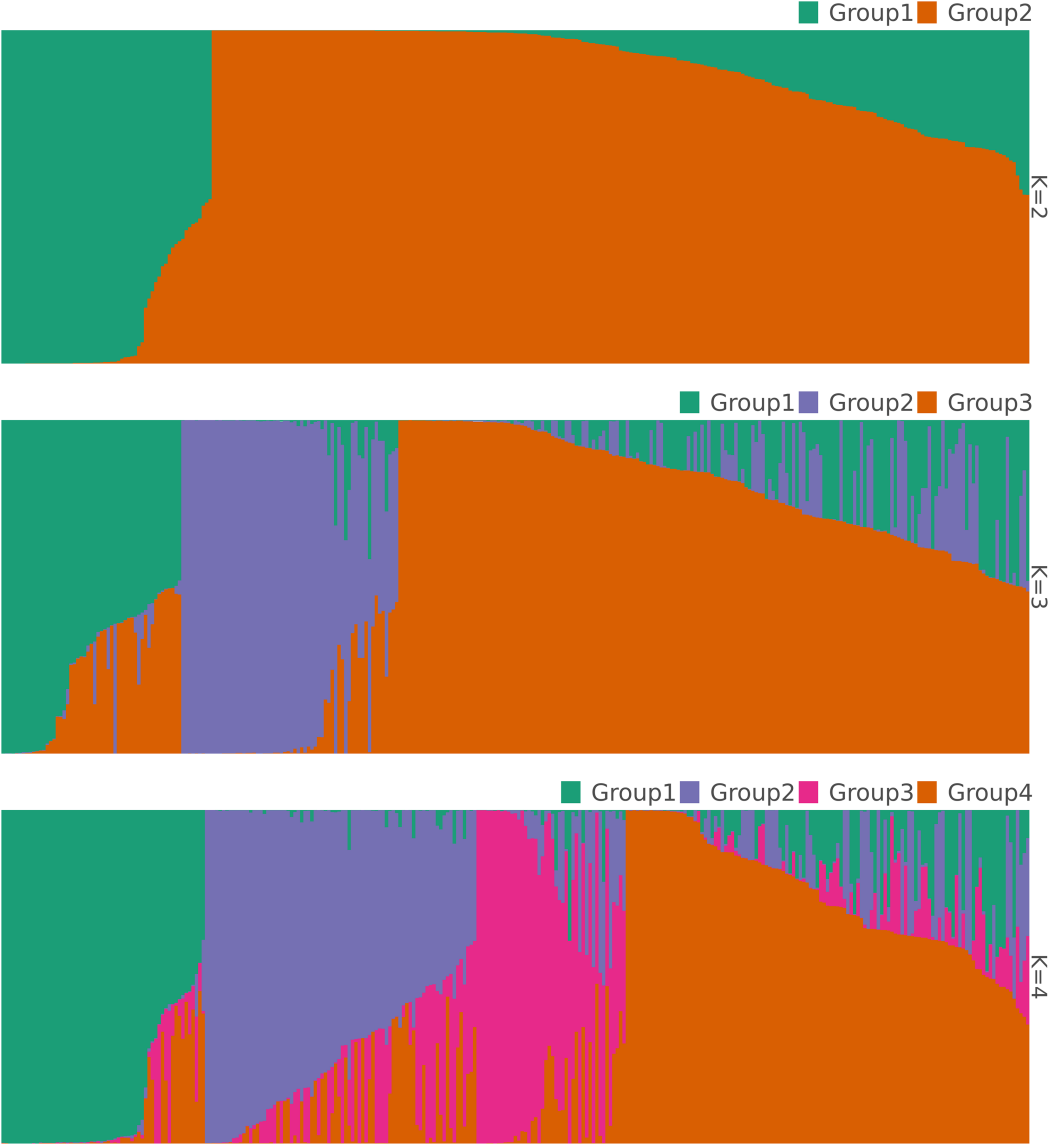
Bar plots showing the structuring pattern of the 305 accessions of jute mallow (*C. olitorius*) based on 1,114 SNPs at K values ranging from 2 to the optimal value of K = 4 clusters.

The result of the analysis of molecular variance based on the 1,114 SNPs from the 305 accessions is presented in **Table 2**. While all sources of variation exerted a highly significant effect (p < 0.001), the difference among accessions within geographical populations accounted for most of the variation (68.3%), whereas only 6% was explained by the difference among the geographical populations. Variation within accessions explained 25.7% of the total variation. The STRUCTURE analysis suggested four optimal clusters (**Supplementary Figure S3**), each encompassing accessions from all the three geographical populations. Seventy-four accessions representing 24% of the studied germplasm were deemed admixed with a membership coefficient threshold of 0.6. Two hundred and thirty-one accessions were categorized in distinct clusters with, 48 accessions (16%), 58 accessions (19%), 28 accessions (9%) and 97 accessions (32%) belonging to genetic group 1, 2 3, 4, respectively (**Figure 3**). The association test (χ2 = 71.623, df = 6, p < 0.001) revealed that geographical origin-wise, the accessions were unevenly distributed across clusters, which may be an indication of a significant dependence between the cluster membership and the accessions geographical origin. For instance, Sudanian zone accessions highly dominated the genetic group 1 while only accessions from the Sudano-Guinean and Sudanian zones formed genetic group 3. Similarly, accessions from the Sudano-Guinean zone were mainly classified into genetic groups 2, 3 and 4 (**Table 3**).

**Table 2:** Results of the hierarchical analysis of molecular variance (AMOVA) based on three phytogeographical zones (Guineo-Congolian, Sudano-Guinean and Sudanian), 305 individual accessions and 1,114 single nucleotide polymorphism (SNP) markers.

Source of variation	Degree of freedom	Sum of squares	Mean square	Variance components	% Variance	P-value
Among phytogeographical zones	2	4144.6	2072.3	9.7	6	< 0.0001
Among accessions within phytogeographical zones	302	78622.8	260.3	109.6	68.3	< 0.0001
Within accessions	305	12568.5	41.20	41.2	25.7	< 0.0001
Total	609	95335.9	156.54	160.5	100	

**Table 3:** Confusion matrix of the assigned genetic groups’ size per phytogeographical region.

Phytogeographical region	Assigned genetic group				Total
	Genetic group 1	Genetic group 2	Genetic group 3	Genetic group 4	
Guineo-Congolian	6	4	2	28	40
Sudano-Guinean	6	43	14	36	99
Sudanian	36	11	12	33	92
Total	48	58	28	97	231

At non-optimal K values of 2 and 3 genetic groups, a total of 11 and 50 admixed accessions were detected, respectively. For both K-values, significant associations were also observed between cluster membership and accessions geographical origin (K = 2: χ2 = 36.40, df = 2, p < 0.001; K = 3: χ2 = 46.5, df = 4, p < 0.001). The Sudanian zone accessions were more sparsely distributed across all different genetic groups compared with accessions from the two other phytogeographical regions that tended at both K = 2 and 3 to be mainly confined to only one genetic cluster.

**Figure 4:**
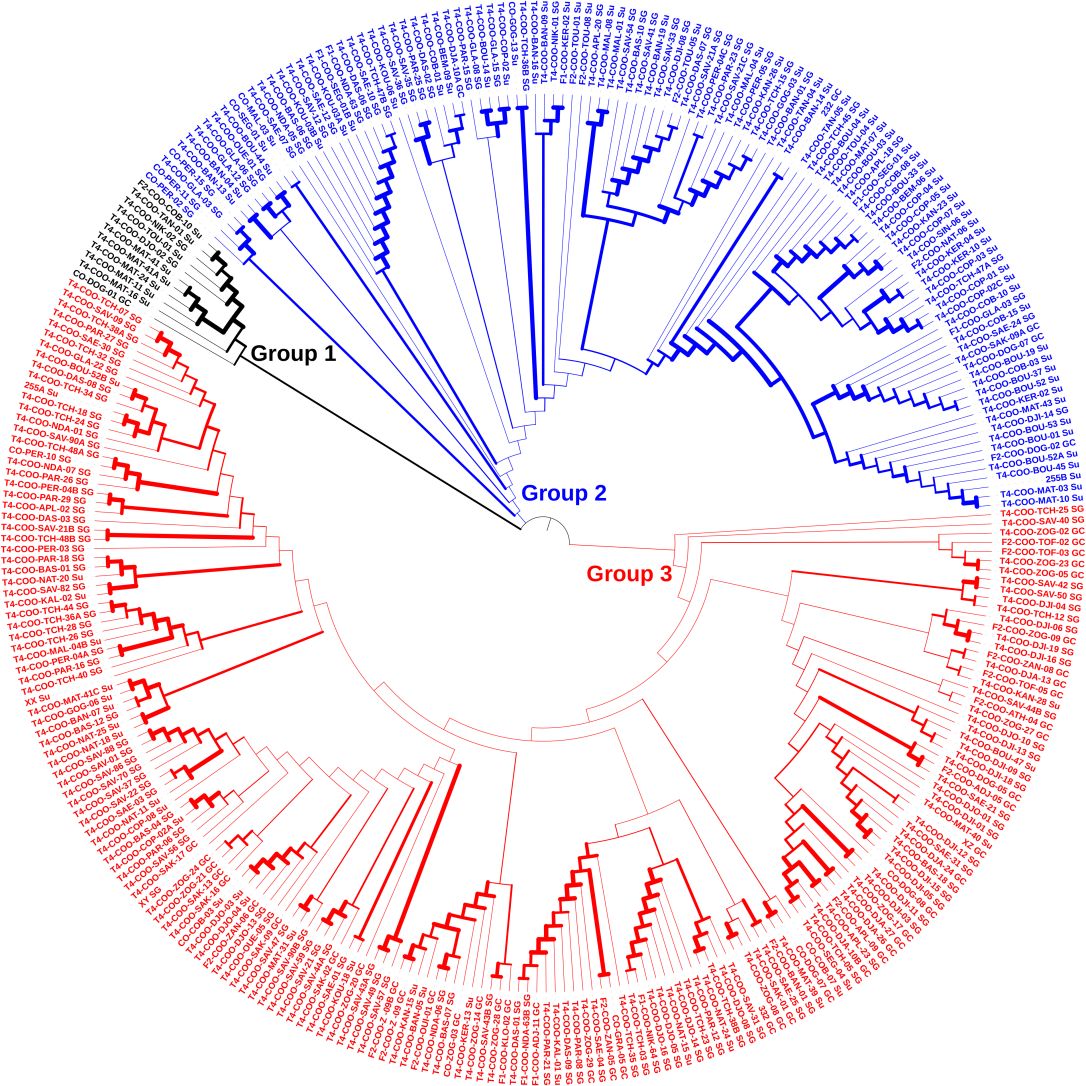
Neighbour joining tree depicting the phylogenetic relationship among the 305 accessions of jute mallow (*C. olitorius*) based on 1,114 SNP markers.

Contrarily to the STRUCTURE analysis, the Neighbour joining (NJ) tree showed three genetic groups (**Figure 4**). The largest group consisted of 181 accessions, intermediate group of 113 accessions, and the smallest group had 11 accessions. This latter was mainly dominated by Sudano-Guinean zone and Sudanian zone accessions. In contrast, accessions of all geographical origins constituted the two other genetic groups.

### 3.4 Core collection development and evaluation

**Figure 5:**
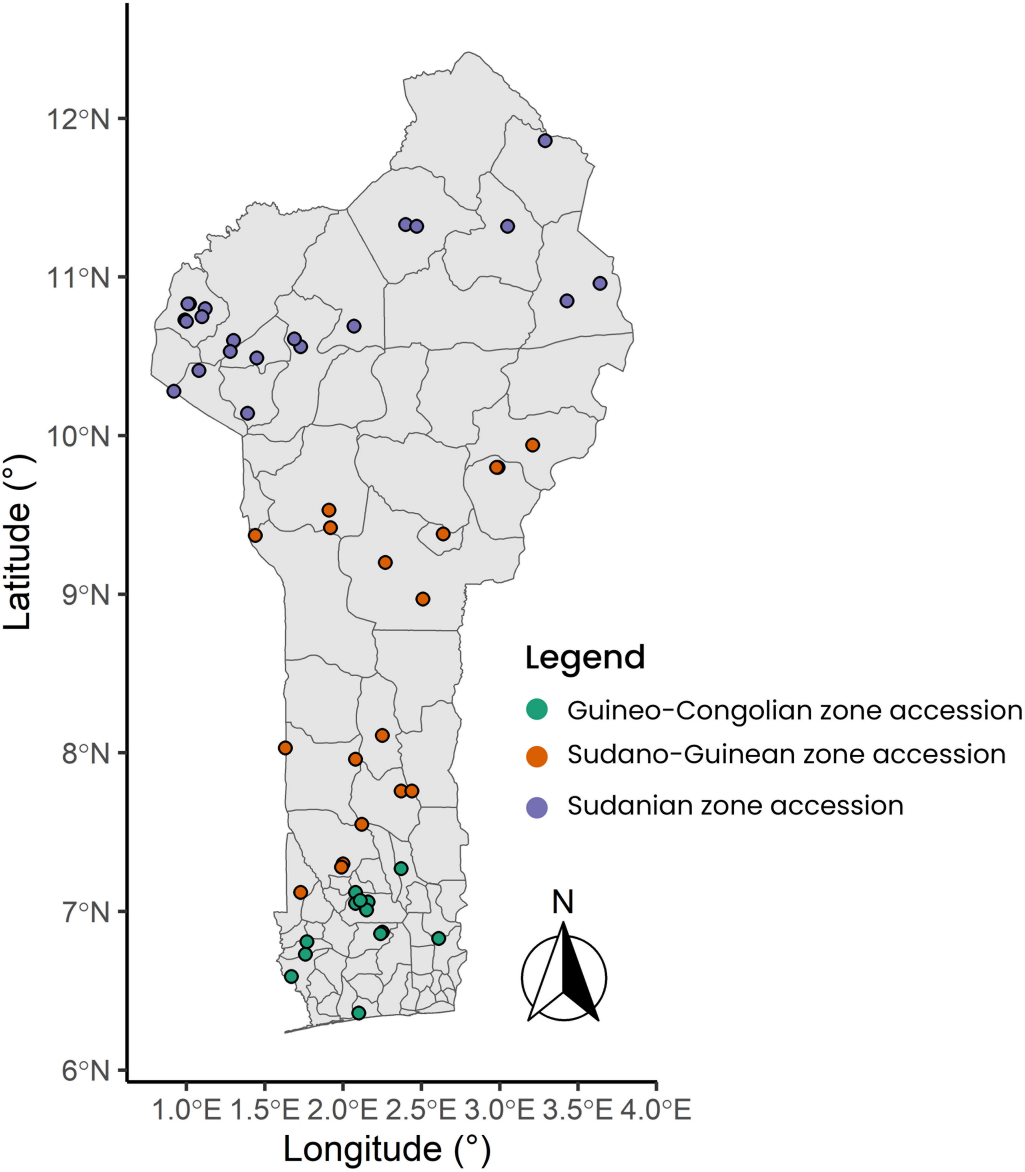
Spatial distribution of the jute mallow *(Corchorus olitorius)* core set accessions based on 1,114 SNP markers analysis.

The ShinyCore algorithm returned a core set composed of 54 accessions representing 17.7% of the total set size. In terms of constitution, this core set was constituted at 24% (13 accessions), 35% (19 accessions) and 41% (22 accessions) of accessions from the Guineo-Congolian (GC), Sudano-Guinean (SG) and Sudanian (Su) geographical populations, respectively (**Figure 5**). These proportions also represented 25.5%, 12.5% and 21.6% of the initial size of the GC, SG and Su populations, respectively. The core set reached a coverage of 98.5% (**Supplementary Figure S4**) for an average accession rarity score of 5.6. A comparison of the diversity statistics between the whole collection and the core collection is shown in **Table 4**. Expected heterozygosity and the total gene diversity estimates in the core set were 0.29 and 0.3, respectively.

**Table 4:** Diversity statistics comparison between the whole and the core collections.

Collection	Diversity statistics		
	Observed heterozygosity	Expected heterozygosity	Total gene diversity
Whole set	0.08 ± 0.001	0.26 ± 0.005	0.28
Core set	0.12 ±	0.29 ±	0.30

### 3.5 Effects of environmental variables on genomic variation

#### 3.5.1 Partial RDA analysis

**Figure 6:**
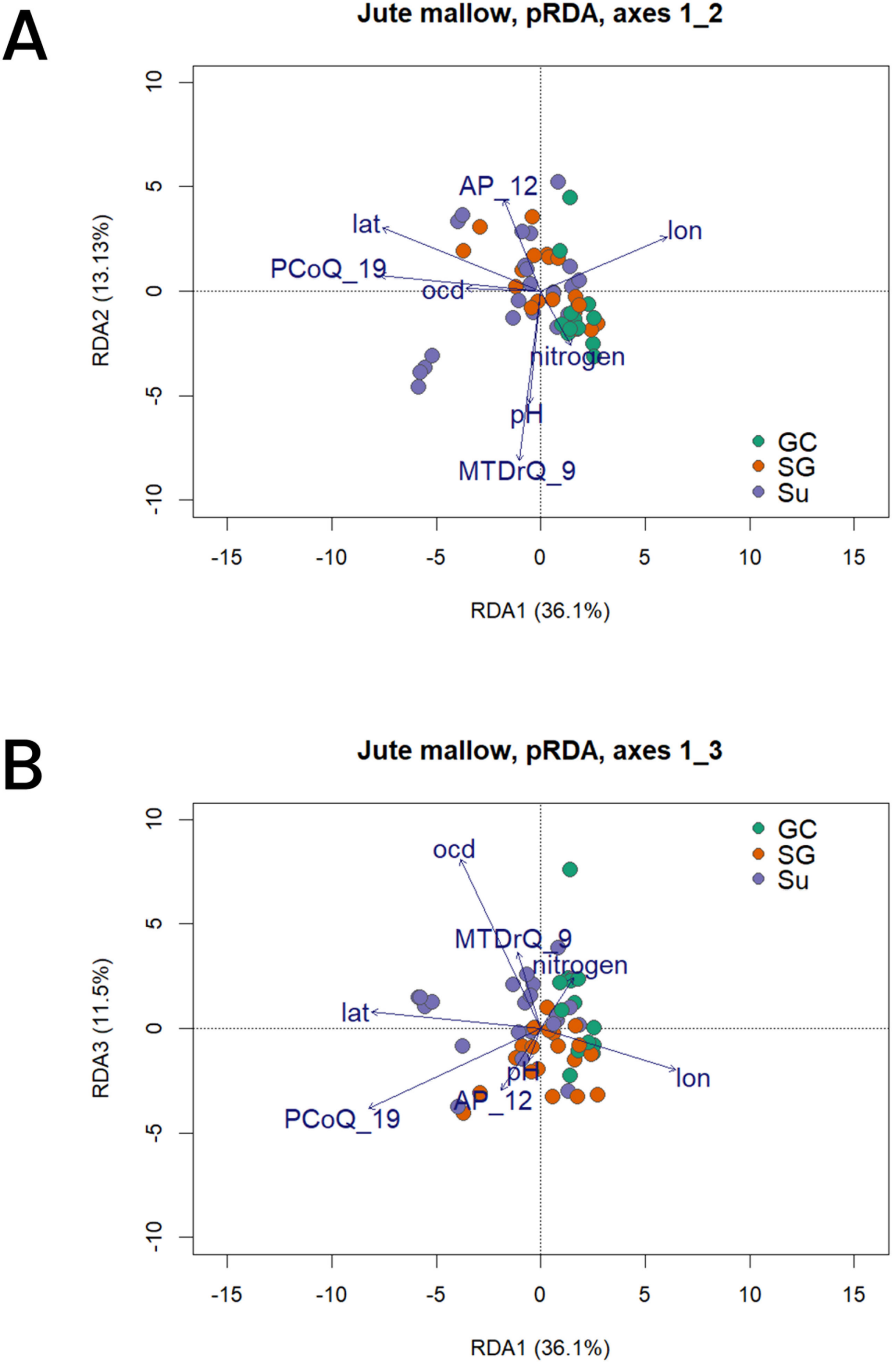
Biplot of the partial RDA (pRDA) conditioned on population structure (axis 1, 2 and 3). The dark blue vectors indicate the direction and value of the associated environmental variables [lat, latitude; lon, longitude; PCoQ_19, precipitation of the coldest quarter; AP_12, annual precipitation; pH, soil water pH; ocd, soil organic carbon density and nitrogen, soil nitrogen content]. **(A)** Core set entries projected on RDA1 and 2, and **(B)** Core set entries projected on RDA1 and 3.

We performed partial RDA accounting for the population structure. We used six environmental variables that were selected as the least correlated and most significant in predicting the genomic variation among the core set entries together with the geographical variables (latitude and longitude). The variables included MTDrQ_9 - mean temperature of the driest quarter; AP_12 - annual precipitation; PCoQ_19 - precipitation of the coldest quarter; pH - soil water pH; ocd - soil organic density; and nitrogen - soil nitrogen content. The first three axes of the pRDA cumulatively accounted for 60.67% of the total variation with axes 1, 2 and 3 explaining 36.08%, 13.13% and 11.46% respectively (**Figures 6A, B**). The population structuring in the partial RDA showed grouping according to populations with the SG and Su populations grouping together while the GC population outgrouped (**Figures 6A, B**). The separation was observed in a latitudinal gradient as is expected due to the ecological gradient in Benin. While the SG and Su populations were positively correlated to the precipitation of the coldest quarter and annual precipitation, the GC was positively correlating to the soil nitrogen content. The observed correlation of the geographic populations and the environmental variables is an indication of their contribution to the population genetic variation geographically and that the variables could be acting as forces driving divergent selection. The highest biplot absolute scores of the first were for longitude (-0.71) and precipitation of the coldest quarter (-0.72) while the lowest was for soil water pH (-0.05). On the second axis, the highest absolute score was observed for mean temperature of the driest quarter (-0.76) and the lowest for soil organic carbon density (0.01).

The pRDA further showed that while the conditioned factor (population structure) accounted for 28.78% of the total variance, the constrained factors (the environmental variables and geography) accounted for 15.23% of the observed genomic variance.

#### 3.5.2 Candidate adaptive SNPs

The four methods detected a total of forty-seven candidate SNPs (**Supplementary Figure S5**). The partial RDA detected the highest number (41) while LFMM and PCAdapt detected 3 and 7 candidate SNPs, respectively. The candidate SNPs detected hardly overlapped except for four between pRDA and PCAdapt methods. Most of the candidate SNPs were associated with mean temperature of the driest quarter (14) and precipitation of the coldest quarter (12). Out of these four SNPs, two were associated to *C. olitorius* specific genes, namely COLO4_08312 and COLO4_12145 and located on chromosome 2 (**Table 5**). COLO4_08312 is linked to oxoacid metabolic processes as well as hydrolyase activity whereas COLO4_12145 is involved in 1- DNA (cytosine-5-)-methyltransferase activity, acting on CpN and CpNpG substrates. One of the SNPs was associated to uncharacterized gene while the other one led to gene involved in the cellular membrane constitution.

**Table 5:** Gene associated with overlapping single nucleotide polymorphism (SNP) detected from the coupled environment association analysis and outlier analysis approaches.

SNP	Chromosome	Gene	Molecular function	Biological process
100217822|F|0-67:T>C-67:T>C	Chr 01 (5577287)	TRIATDRAFT_251069	Uncharacterized	–
100194604|F|0-50:C>T-50:C>T	Chr 02 (37034568)	COLO4_08312	Oxoacid metabolic process; hydrolyase activity	Stress (drought) resistance
100161554|F|0-12:G>T-12:G>T	Chr 02 (48332274)	COLO4_12145	1- DNA (cytosine-5-)-methyltransferase activity, acting on CpN and CpNpG substrates; DNA binding	Methylation; negative regulation of gene expression via chromosomal CpG island methylation
100180864|F|0-44:C>G-44:C>G	Chr 03 (10333947)	D3D02_12115	Cellular membrane constitution	–

#### 3.5.3 Fitness of the core set in the future climate scenario

**Figure 7:**
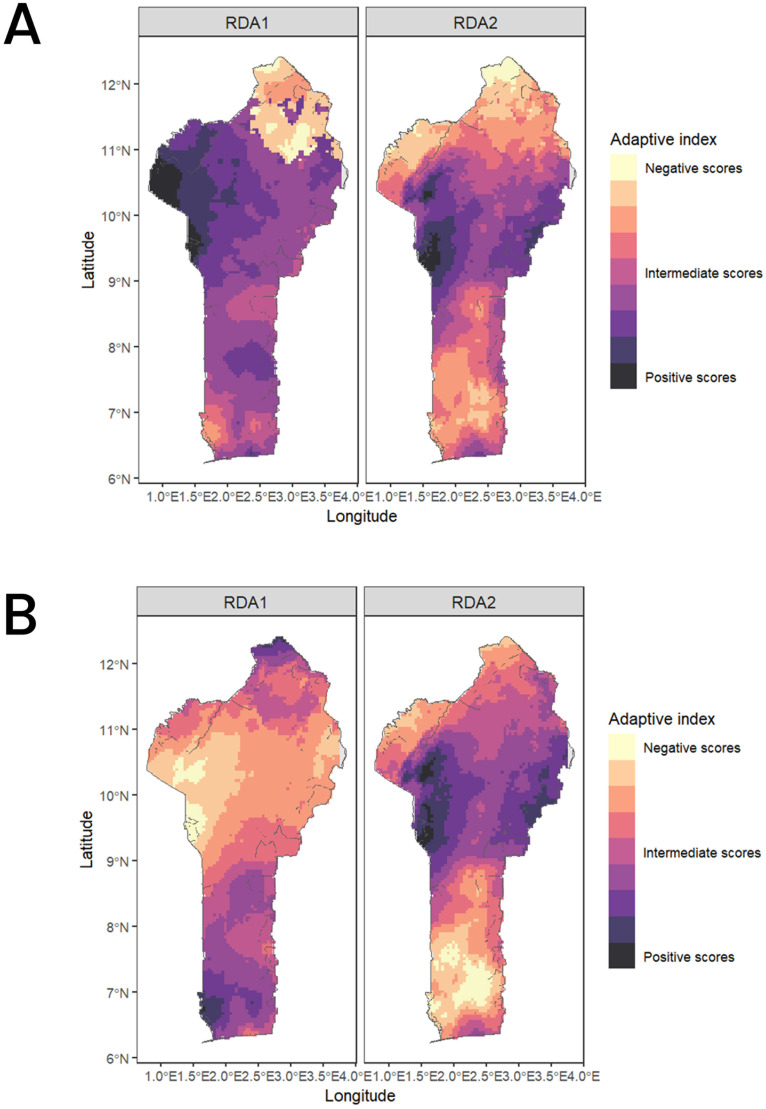
Adaptive space for RDA1 and RDA2 along the Benin ecological for the current climate conditions **(A)** and the future climatic conditions **(B)**.

**Figure 8:**
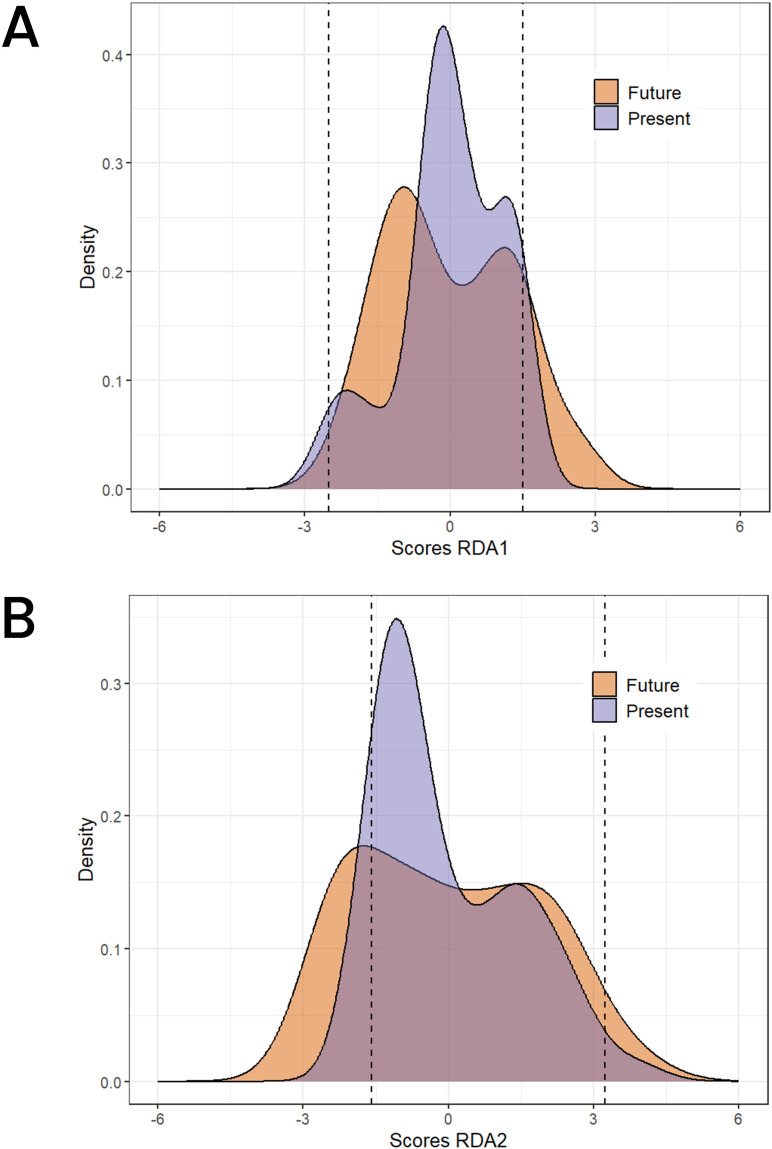
Adaptive space for RDA1 and for the current climate conditions **(A)** and the future climatic conditions **(B)**. The dashed line represents the 95% interval of the RDA scores distribution.

The future predictions of 2061–2080 using the two SSP scenarios shows a concerning change for the northern part of the SG zones and the Su zones on RDA1 (**Figure 7A**) and for the GC zone on RDA2 (**Figure 7B**) as revealed by a change to lighter pixels from current to future climatic conditions. Overall, we do not observe a strong change of the adaptive score on RDA2 except for the southern regions where some environments are shifting to more negative values. Most of the occurrences (95% shown by the dashed lines in **Figure 8**) experience negative scores in RDA1 compared to RDA2 for both present and future conditions. Generally, we observed a shift towards the negative values on both RDA1 and RDA2 indicated by the skewness of the adaptive scores in the future climates compared to the case in the current conditions (**Figure 8**).

**Figure 9:**
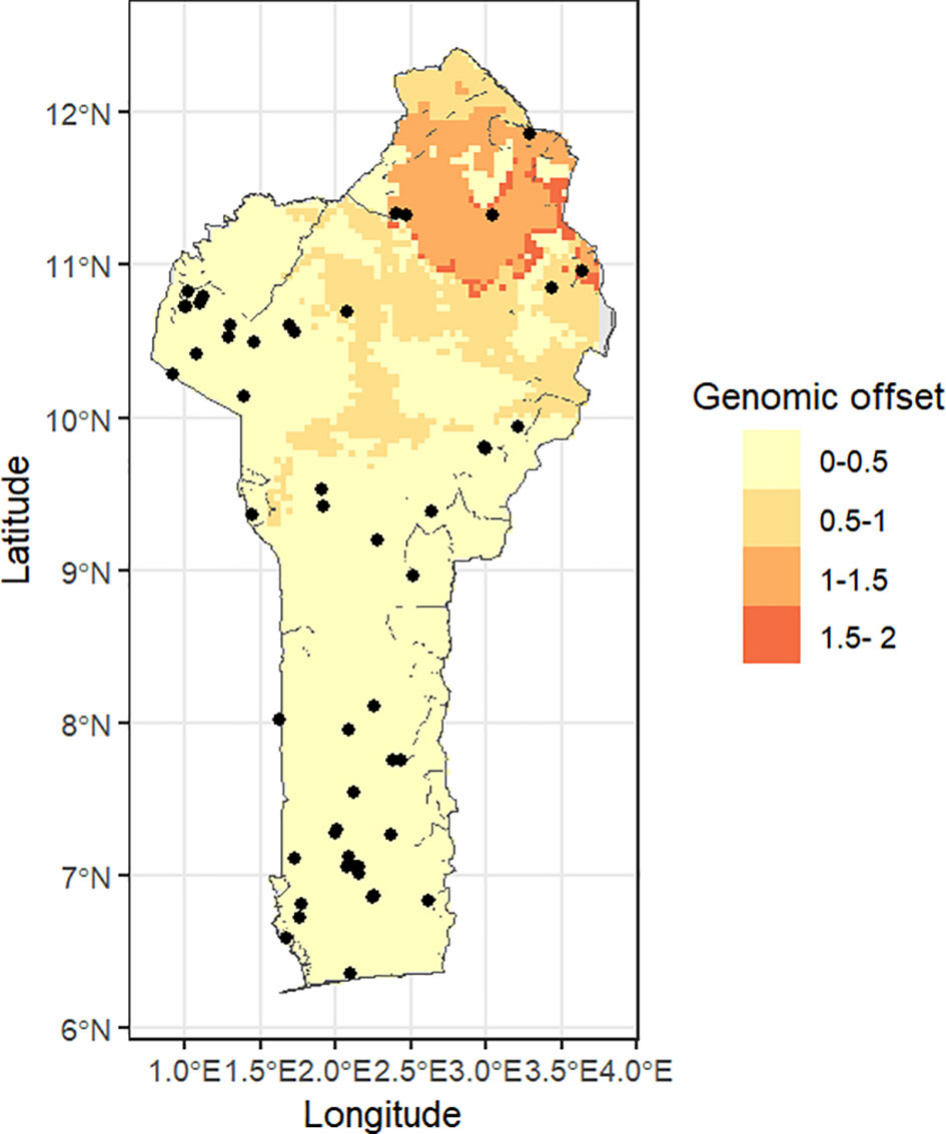
Spatial distribution of genomic offset estimated under the Shared Socieconomic Pathways 370 (SSP370) and the Shared Socieconomic Pathways (585) for 2061-2080.

Finally, we build a global spatial distribution of the genomic offset between the current adaptive scores and the future conditions. In the scenario for future climate change, we observed a pattern of lower genomic offset predictions for population in the GC in the southern region compared to the SG and Su populations towards the north (**Figure 9**).

## 4 Discussion

This study generates the first genomic resources for *Corchorus olitorius* accessions in Benin, and opens the rooms for in-depth genebank genomics studies. It revealed how genomic diversity in *C. olitorius* evolved along the south-north ecological gradient in Benin as well as the difference among accessions among and within geographical populations. A core collection was set and exhibit similar genetic parameters with the whole population. Moreover, the genomic structure of the core set was co-analyzed with geographical, climatic and pedological parameters to understand how the derived core set population can adapt to environmental conditions changes.

### 4.1 Markers quality and genomic diversity in *Corchorus olitorius*

Quality markers are important in genomics analyses. Among the most used post-filtering quality control parameters is the Ts/Tv ratio, with a high value of this parameter translating a high SNP quality as long as the value is < 4 (Wang et al., 2015). The 1,114 SNPs employed in this study exhibited a Ts/Tv ratio value (1.4) that fitted in the range [1, 4]. Our markers therefore, can, be inferred to be of good quality. This number of SNP used also compared well with the 1,115 RADSeq SNP markers used by Sarkar et al. (2019) to delineate African and Asian populations of *C. olitorius*, for which a Ts/Tv ratio value of 1.6 was reported.

Regarding informativeness, PIC is a standard parameter for translating markers usefulness in conveying polymorphism information. Its value ranges from 0 to 0.5 in biallelic markers such as those used in this study, and from 0 to 1 in multi-allelic markers such as single sequence repeat markers (SSR) and AFLP. Against this background, the 1,114 markers used to characterize *C. olitorius* with their average PIC of 0.27 can be deemed highly informative when compared to markers used in the assessment of other vegetable crops diversity such as Amaranth (*Amaranthus* spp.) (Jamalluddin et al., 2022), snake melon (*Cucumis melo* L.) (Abu Zaitoun et al., 2018), cultivated and wild tomato (*Solanum lycopersicum* L. and *S. pimpinellifolium* L.) (Ayenan et al., 2021) for which PIC values fell in the range of 0.14 - 0.23.

The core knowledge so far available on the molecular diversity in *C. olitorius* was based on single sequence repeat (SSRs) markers and has put forward an overall low to moderate diversity in *C. olitorius* (Adeyemo et al., 2021; Benor et al., 2012; Ghosh et al., 2017; Roy et al., 2006; Yang et al., 2018). Apart from the study that reported on the reference genome of *C. olitorius*, the only available study employing next generation sequencing to study diversity in the species also concluded on a low diversity in the species (Sarkar et al., 2019). Here, we provided the first report of DArTseq SNP-based genome-wide diversity analysis on Benin *C. olitorius* accessions sampled from the worldwide largest collection of the species held at CalaviGen. Our findings rather supported a moderate diversity in the species, pinpointing the collection as a valuable resource that breeders could tap in for various improvement needs. *Corchorus olitorius* being a predominantly self-pollinated crop, one might expect a low diversity in the species (Huang et al., 2009). The moderate diversity exemplified by the expected heterozygosity estimate of 0.27 in this study is specific since this parameter strongly depended on the combination of the used germplasm and the set of single nucleotide polymorphism markers considered. The population genomics analysis conducted in this study revealed a strong difference in the population’s genome-wide diversity with the heterozygosity estimates decreasing following the North-South ecological gradient of Benin regions. This echoed a latitudinal variation pattern in the *C. olitorius* diversity organization in the species in Benin and opened the room for a proper investigation of the influence that some discriminating climatic variables among the phytogeographical zones (e.g., precipitation, relative humidity) may have on the species. Although the prominent uses of SNPs marker in population genomics is the calculation of heterozygosity estimates at both the species and population levels (Schmidt et al., 2024), such a practice has started raising concerns over the recent years. According to Schmidt et al. (2021) because SNP-based heterozygosity estimates only integrated polymorphic sites, their value is always affected by the global sample size (n), with smaller sample sizes producing larger heterozygosity estimates. This render, in the sense of these authors, trivial and arguable the comparison of SNP heterozygosity estimates across studies on the same species or among species. A suggestion to cope with this limitation has been the introduction of the autosomal heterozygosity that integrated the number of invariable sites in the heterozygosity. Consequently, the herewith presented population level heterozygosity estimates do not warrant being involved in any comparison with any other studies or to any other species. In lieu and place, the correct estimates to consider for such comparisons is the autosomal heterozygosity whose value is always lower than the SNP heterozygosity. In this study, the trend suggested by the autosomal heterozygosity estimates across the various populations perfectly aligned with the one painted by the SNP heterozygosity thus reinforcing the north-south declining latitudinal variation in the species diversity. The relatively highest diversity of *C. olitorius* in the Sudanian zone was further accompanied by a high allelic richness, which together indicate that this population should be given priority when it comes to conservation. The lower autosomal diversity recorded in the Guineo-Congolian seemed to reflect a high selection pressure imposed by the south Benin inhabitants, a region highly specialized in the species cultivation (Link 3) and where the search of performant materials may have led to the unconscious narrowing of the diversity.

### 4.2 Differentiation and *Corchorus olitorius* population structure

Despite the distinct diversity level observed among the three populations, these populations appeared poorly differentiated. This suggested a high gene flow among the populations certainly that could have resulted from a possible material exchange among producers, a practice commonly reported in any informal seed system (Bèye and Wopereis, 2014), which is still prominent in the case of *C. olitorius* in Benin. It is expected from annual self-pollinated species to retain high variation among populations and to present a low diversity coupled with a high differentiation while cross-pollinated species are known to exhibit high variation within population, low differentiation, and a high diversity (Andriamihaja et al., 2021; Tchokponhoué et al., 2023). In this study, the highest molecular variation was detected among accessions within populations while differentiation was low and diversity moderate. Such a trend seemed to echo an equal prominence of outcrossing in *C. olitorius* that was up to now seen as a predominantly self-pollinated species. This hypothesis was further strengthened by the relatively high admixture rate (48/305 accessions) observed in the collection. Given the high diversity reported in the Sudanian zone and considering the fact that the STRUCTURE-derived genetic group 1 (made up of 48 accessions) was mainly made-up of accessions from the same region, we then suggested this group 1 to be of high interest for conservation.

### 4.3 Core set quality

Plant genetic resources genebanking has been reported crucial in modern agriculture endeavour to limiting genetic erosion (Aubry, 2023). In such a context, the development of core collection has been put forward to reduce germplasm diversity complexity for enhanced conservation. Although core collection was developed for a diversity of vegetables (Lee et al., 2016; Schafleitner et al., 2021, 2022), *C. olitorius* has so far missed this precious working asset. In this study we derived the first ever known core collection for the jute mallow considering accessions from the world largest germplasm holder of the species. This extracted core represented 17.4% of the whole germplasm sizestudied and fitted very well within the recommended size of 10-30% (of the entire collection) for a good collection (Bhattacharjee et al., 2007; Tchokponhoué et al., 2020). Interestingly, the core set developed painted a nice representativeness of all the three geographical populations studied. Depending on the approach, several indicators were proposed to gauge the quality of a core collection. These included for instance large variance difference, variable rate and coincidence range in the case on the phenotypic data-based approach (Mahmoodi et al., 2019; Risliawati et al., 2023) while maximal expected heterozygosity, maximal total gene diversity and high coverage are expected when it comes to SNP-based core collection, and especially the CCI-type one (Kim et al., 2023; Marita et al., 2000; Odong et al., 2013). In this SNP-based study, the diversity estimates for both the observed and expected heterozygosity as well as for the total gene diversity were all higher in the core set than in the whole collection. As for the coverage (CV), although reaching the value of 99 - 100% is desired (Jeong et al., 2017), the ShinyCore algorithm used in this study allowed us to seek for a trade-off between losing a slight CV at the cost of harvesting more rare alleles. Here, the core set obtained had a coverage of 98.5% which is also close to 99%, allowing including accessions whose average rarity score was 5.6. At 48 accessions extracted, the core set coverage asymptote was already reached. This allowed for the inclusion in our coreset of the top six accessions exhibiting the greatest allele rarity score. Such rare accessions are of particular interest for both conservation and breeding. Taken together, these criteria suggested that the core set developed is robust enough to represent the studied germplasm and can therefore be involved in finer studies. In this vein, next stages should then encompass discovering the agronomic and nutritional value of this core set.

### 4.4 Potential of the *C. olitorius* core collection in the future ecosystem in Benin

Under a changing environment, the future of the extracted core set accessions may be questionable. Future climate predictions indicate that there will be significant changes in the rainfall (Ahokpossi, 2018) and temperature patterns, an observation that the various adaptive spaces generated in this work corroborated. This will definitely affect the farming ecosystems across Africa (Tefera et al., 2025). In our study, we employ a novel approach of applying landscape genomics analysis to find insights on the likely shifts in fitness that the diversity in our jute mallow core collection might experience under the future climate conditions. Overall, our model shows that the fitness of our core collection would not be severely impacted except for a few entries originating from the northern regions according to the three model scenarios used in this analysis. Indeed, all the adaptive SNP detected were associated with genes involved in biological processes pertaining to abiotic stress, in particular drought, resistance regulation. For instance the oxoacid metabolic process encoded by the gene COLO4_08312 was reported to modulate resistance to water scarcity in maize (*Zea mays* L.) (Huet al., 2024) and in tomato (*Solanum lycopersicum* and *S. peruvianum* L.) (Tapia et al., 2021). Indeed, the oxoacid metabolic process encompassed genes linked to the biosynthesis of gibberellin and abscisic acid, two hormones known to be involved in plant adaptation to abiotic stresses such as salt and drought, among others (Colebrook et al., 2014; Sah et al., 2016). These northern regions also fall in the higher altitude regions of Benin, corroborating a fact that predictions have placed special concern of climate change effects especially in the high-altitude ecosystems (Wang et al., 2024). Going by the spatial modelling of jute mallow fitness within the landscape in Benin, special concern should be placed on the northern populations and perhaps building a core collection that incorporates new collections from the region from time to time would be recommended. The effects of the future climate shifts may also change overtime depending on the closeness of the populations to available adaptive alleles in the nearby populations within the dispersal range and also due to the fact that jute mallow is a short season crop. The risk of maladaptation can also be mitigated by assisting gene flow through farmer exchange to enable introduction of adaptive alleles into the populations that would require the greatest change in their adaptive genetic capacity for the future conditions.

This study provides valuable information on the measure of the standing adaptive variation present in a wide collection of the jute mallow in Benin by integrating the environmental association analysis. For now, the fifty-four entries of the core collection look promising to tap into for the improvement of the jute mallow in Benin region.

## 5 Conclusion

This work employed single nucleotide polymorphism to profile the genome wide diversity variation in a vegetable crop along the natural north-south ecological gradient in Benin. It offered a clear view of how the South-North ecological gradient shaped genomic diversity in the country-wide *C. olitorius* collection held at CalaviGen. Based on 1,114 high quality SNPs we demonstrated that diversity was higher in the Sudanian zone although the three populations studied were poorly differentiated. The 305 accessions studied were classified into three to four genetic groups, and a core set of 54 accessions carrying out the diversity found within the whole germplasm was derived and proposed as a candidate core collection for germplasm exchange in forthcoming collaborative initiatives. The low genomic offset exhibited by most of the 54 accessions placed this core collection as a valuable resource to conserve and to promote for cultivation in the context of changing environment.

## Data Availability

The datasets presented in this study can be found in online repositories. The names of the repository/repositories and accession numbers can be found in the article/**Supplementary Material**.

## References

[B1] Abu Zaitoun S. Y. Jamous R. M. Shtaya M. J. Mallah O. B. Eid I. S. Ali-Shtayeh M. S. (2018). Characterizing Palestinian snake melon (*Cucumis melo* var. *flexuosus*) germplasm diversity and structure using SNP and DArTseq markers. BMC Plt. Biol. 18, 1–12. doi: 10.1186/s12870-018-1475-2, PMID: 30340523 PMC6194588

[B2] Achigan-Dako E. G. Pasquini M. W. Assogba Komlan F. N’danikou S. Yédomonhan H. Dansi A.. (2010). Traditional vegetables in Benin (Cotonou: Institut National des Recherches Agricoles du Bénin).

[B3] Adebo H. O. Ahoton L. E. Quenum F. Ezin V. (2015). Agro-morphological characterization of *Corchorus olitoriu*s cultivars of Benin. Ann. Res. Rev. Biol. 7, 229–240. doi: 10.9734/ARRB/2015/17642

[B4] Adeyemo O. A. Ayodele O. O. Ajisafe M. O. Okinedo U. E. Adeoye D. O. Afanou A. B.. (2021). Evaluation of dark jute SSR markers and morphological traits in genetic diversity assessment of jute mallow (*Corchorus olitorius* L.) cultivars. S. Afr. J. Bot. 137, 290–297. doi: 10.1016/j.sajb.2020.10.027

[B5] Adjatin A. Loko Yêyinou E. L. Zaki B. Balogoun D. Odjo T. Yedomonhan H.. (2019). Agromorphological characterization of jute (*Corchorus olitorius* L.) landraces in Central region of Benin Republic. Int. J. Adv. Res. Biol. Sci. 6, 96–107. doi: 10.22192/ijarbs.2019.06.11.013

[B6] Afokpe P. M. K. Ologou S. Kouiho S. R. de Hoop S. J. N’Danikou S. Achigan-Dako E. G.. (2025). Unveiling genetic diversity in jute mallow (*Corchorus* spp.): morphological clustering reveals distinctive traits among accessions from Africa and Asia. Genet. Resour. Crop Evol. 72, 5753–5775. doi: 10.1007/s10722-024-02295-7

[B7] Ahmed F. (2021). Nutraceutical potential of molokhia (Corchorus olitorius L.): A versatile green leafy vegetable. Pharmacogn. Res. 13, 1–12. doi: 10.4103/pr.pr_100_20

[B8] Ahokpossi Y. (2018). Analysis of the rainfall variability and change in the Republic of Benin (West Africa). Hydrol. Sci. J. 63, 2097–2123. doi: 10.1080/02626667.2018.1554286

[B9] Andriamihaja C. F. Ramarosandratana A. V. Grisoni M. Jeannoda V. H. Besse P. (2021). Drivers of population divergence and species differentiation in a recent group of indigenous orchids (*Vanilla* spp.) in Madagascar. Ecol. Evol. 11, 2681–2700. doi: 10.1002/ece3.7224, PMID: 33767829 PMC7981232

[B10] Aubry S. (2023). Genebanking plant genetic resources in the postgenomic era. Agr. Hum. Values 40, 961–971. doi: 10.1007/s10460-023-10417-7

[B11] Ayenan M. A. T. Danquah A. Agre P. A. Hanson P. Asante I. K. Danquah E. Y. (2021). Genomic and phenotypic diversity of cultivated and wild tomatoes with varying levels of heat tolerance. Genes 12, 503. doi: 10.3390/genes12040503, PMID: 33805499 PMC8067180

[B12] Benor S. Demissew S. Hammer K. Blattner F. R. (2012). Genetic diversity and relationships in *Corchorus olitorius* (Malvaceae) inferred from molecular and morphological data. Genet. Resour. Crop Evol. 59, 1125–1146. doi: 10.1007/s10722-011-9748-8

[B13] Bèye A. M. Wopereis M. C. S. (2014). Cultivating knowledge on seed systems and seed strategies: case of the rice crop. Net J. Agric. Sci. 2, 11–29.

[B14] Bhattacharjee R. Khairwal I. S. Bramel P. J. Reddy K. N. (2007). Establishment of a pearl millet [*Pennisetum glaucum* (L.) R. Br.] core collection based on geographical distribution and quantitative traits. Euphytica 155, 35–45. doi: 10.1007/s10681-006-9298-x

[B15] Burkill H. M. (2004). The useful plants of West Tropical Africa Vol. 6 (Kew: Royal Botanical Gardens).

[B16] Capblancq T. Forester B. R. (2021). Redundancy analysis: A Swiss Army Knife for landscape genomics. Methods Ecol. Evol. 12, 2298–2309. doi: 10.1111/1755-0998.12906, PMID: 29802785

[B17] Capblancq T. Luu K. Blum M. G. Bazin E. (2018). Evaluation of redundancy analysis to identify signatures of local adaptation. Mol. Ecol. Res. 18, 1223–1233. doi: 10.1111/1755-0998.12906, PMID: 29802785

[B18] Caye K. Jumentier B. Lepeule J. François O. (2019). LFMM 2: fast and accurate inference of gene-environment associations in genome-wide studies. Mol. Biol. Evol. 36, 852–860. doi: 10.1093/molbev/msz008, PMID: 30657943 PMC6659841

[B19] Christov N. K. Tsonev S. Todorova V. Todorovska E. G. (2021). Genetic diversity and population structure analysis–a prerequisite for constructing a mini core collection of Balkan *Capsicum annuum* germplasm. Biotechnol. Biotechnol. Equip. 35, 1010–1023. doi: 10.1080/13102818.2021.1946428

[B20] Colebrook E. H. Thomas S. G. Phillips A. L. Hedden P. (2014). The role of gibberellin signalling in plant responses to abiotic stress. J. Exp. Biol. 217, 67–75. doi: 10.1242/jeb.089938, PMID: 24353205

[B21] Coulon A. Fitzpatrick J. Bowman R. Stith B. Makarewich C. Stenzler L.. (2008). Congruent population structure inferred from dispersal behaviour and intensive genetic surveys of the threatened Florida scrub-jay (*Aphelocoma coerulescens*). Mol. Ecol. 17, 1685–1701. doi: 10.1111/j.1365-294X.2008.03705.x, PMID: 18371014

[B22] De Beukelaer H. Davenport G. F. Fack V. (2018). Core Hunter 3: flexible core subset selection. BMC Bionformatics 19, 1–12. doi: 10.1186/s12859-018-2209-z, PMID: 29855322 PMC6092719

[B23] Evanno G. Regnaut S. Goudet J. (2005). Detecting the number of clusters of individuals using the software STRUCTURE: a simulation study. Mol. Ecol. 14, 2611–2620. doi: 10.1111/j.1365-294X.2005.02553.x, PMID: 15969739

[B24] Fick S. E. Hijmans R. J. (2017). WorldClim 2: new 1-km spatial resolution climate surfaces for global land areas. Int. J. Climatol. 37, 4302–4315. doi: 10.1002/joc.5086

[B25] Fitzpatrick M. C. Keller S. R. (2015). Ecological genomics meets community-level modelling of biodiversity: Mapping the genomic landscape of current and future environmental adaptation. Ecol. Lett. 18, 1–16. doi: 10.1111/ele.12376, PMID: 25270536

[B26] Francis R. M. (2017). pophelper: an R package and web app to analyse and visualize population structure. Mol. Ecol. Res. 17, 27–32. doi: 10.1111/1755-0998.12509, PMID: 26850166

[B27] Frankel O. H. (1984). “Genetic perspectives of germplasm conservation,” in Genetic Manipulation: Impact on Man and Society. Eds. Arber W. K. Illmensee K. Peacock W. J. Starlinger P. Ehrlich S. D. Dagert M. Romac S. Michel B. Levy S. B. Goebel W. (Cambridge University Press, Cambridge, Cambridge, UK), 161–170.

[B28] Ghamkhar K. Snowball R. Wintle B. Brown A. (2008). Strategies for developing a core collection of bladder clover (*Trifolium* sp*umosum* L.) using ecological and agro-morphological data. Aust. J. Agric. Res. 59, 1103–1112. doi: 10.1071/AR08209

[B29] Ghosh S. Meena K. Sinha M. Karmakar P. (2017). Genetic diversity in *Corchorus olitorius* genotypes using jute SSRs. Proc. Natl. Acad. Sci. India Sect. B Biol. Sci. 87, 917–926. doi: 10.1007/s40011-015-0652-4

[B30] Girma G. Tirfessa A. Bejiga T. Seyoum A. Mekonen M. Nega A.. (2024). Assessing genetic, racial, and geographic diversity among Ethiopian sorghum landraces and implications for heterotic potential for hybrid sorghum breeding. Mol. Breed. 44, 46. doi: 10.1007/s11032-024-01483-8, PMID: 38911335 PMC11190104

[B31] Gruber B. Georges A. Mijangos J. L. Pacioni C. Unmack P. J. Berry O. (2023). Analysing ‘SNP’ and ‘Silicodart’ data - basic functions.

[B32] Gu R. Fan S. Wei S. Li J. Zheng S. Liu G. (2023). Developments on Core collections of plant genetic resources: do we know enough? Forests 14, 926. doi: 10.3390/f14050926

[B33] Hemstrom W. Jones M. (2023). snpR: User friendly population genomics for SNP data sets with categorical metadata. Mol. Ecol. Res. 23, 962–973. doi: 10.1111/1755-0998.13721, PMID: 36239472

[B34] Hengl T. Mendes de Jesus J. Heuvelink G. B. Ruiperez Gonzalez M. Kilibarda M. Blagotić A.. (2017). SoilGrids250m: Global gridded soil information based on machine learning. PloS One 12, e0169748. doi: 10.1371/journal.pone.0169748, PMID: 28207752 PMC5313206

[B35] Hijmans R. J. Bivand R. Dyba K. Pebesma E. Sumner M. (2023). Terra [R Package]. R Programming Language.

[B36] Hong J.-P. Ro N. Lee H.-Y. Kim G. W. Kwon J.-K. Yamamoto E.. (2020). Genomic selection for prediction of fruit-related traits in pepper (*Capsicum* spp.). Front. Plt. Sci. 11. doi: 10.3389/fpls.2020.570871, PMID: 33193503 PMC7655793

[B37] Hu X. Cheng J. Lu M. Fang T. Zhu Y. Li Z.. (2024). Ca2+-independent ZmCPK2 is inhibited by Ca2+-dependent ZmCPK17 during drought response in maize. J. Integr. Plant Biol. 66, 1313–1333. doi: 10.1111/jipb.13675, PMID: 38751035

[B38] Huang Y. Zhang C. Q. Li D. Z. (2009). Low genetic diversity and high genetic differentiation in the critically endangered *Omphalogramma souliei* (Primulaceae): implications for its conservation. J. Syst. Evol. 47, 103–109. doi: 10.1111/j.1759-6831.2009.00008.x

[B39] Jamalluddin N. Massawe F. J. Mayes S. Ho W. K. Symonds R. C. (2022). Genetic diversity analysis and marker-trait associations in Amaranthus species. PloS One 17, e0267752. doi: 10.1371/journal.pone.0267752, PMID: 35551526 PMC9098028

[B40] Jeong S. Kim J.-Y. Jeong S.-C. Kang S.-T. Moon J.-K. Kim N. (2017). GenoCore: A simple and fast algorithm for core subset selection from large genotype datasets. PloS One 12, e0181420. doi: 10.1371/journal.pone.0181420, PMID: 28727806 PMC5519076

[B41] Kakpo A. B. Ladekan E. Y. Dassou H. Gbaguidi F. Kpoviessi S. Gbenou J. D. (2019). Ethnopharmacological investigation of medicinal plants used to treat typhoid fever in Benin. J. Pharmacogn. Phytochem. 8, 225–232.

[B42] Kamvar Z. N. Tabima J. F. Everhart S. E. Brooks J. C. Krueger-Hadfield S. A. Sotka E. (2024). Poppr: an R package for genetic analysis of populations with clonal, partially clonal, and/or sexual reproduction. PeerJ 2, 1–14. doi: 10.7717/peerj.281, PMID: 24688859 PMC3961149

[B43] Kang Y. Choi C. Kim J. Y. Min K. D. Kim C. (2023). Optimizing genomic selection of agricultural traits using K-wheat core collection. Front. Plt Sci. 14. doi: 10.3389/fpls.2023.1112297, PMID: 37389296 PMC10303932

[B44] Kim S. Kim D. S. Moyle H. Heo S. (2023). ShinyCore: An R/Shiny program for establishing core collection based on single nucleotide polymorphism data. Plant Methods 19, 106. doi: 10.1186/s13007-023-01084-0, PMID: 37821997 PMC10566191

[B45] Kirby R. H. (1963). Vegetable fibres, botany, cultivation and utilization.

[B46] Lee H.-Y. Ro N.-Y. Jeong H.-J. Kwon J.-K. Jo J. Ha Y.. (2016). Genetic diversity and population structure analysis to construct a core collection from a large Capsicum germplasm. BMC Genet. 17, 1–13. doi: 10.1186/s12863-016-0452-8, PMID: 27842492 PMC5109817

[B47] Legendre P. Legendre L. (2012). “Canonical analysis,” in Developments in environmental modelling, vol. 24. (United Kingdom: Elsevier), 625–710.

[B48] Letunic I. Bork P. (2024). Interactive Tree of Life (iTOL) v6: recent updates to the phylogenetic tree display and annotation tool. Nucleic Acids Res. 52, W78–W82. doi: 10.1093/nar/gkae268, PMID: 38613393 PMC11223838

[B49] Luu K. Bazin E. Blum M. G. (2017). pcadapt: an R package to perform genome scans for selection based on principal component analysis. Mol. Ecol. Res. 17, 67–77. doi: 10.1111/1755-0998.12592, PMID: 27601374

[B50] Mahalakshmi V. Ng Q. Lawson M. Ortiz R. (2007). Cowpea [*Vigna unguiculata* (L.) Walp.] core collection defined by geographical, agronomical and botanical descriptors. Plant Genet. Res. 5, 113–119. doi: 10.1017/S1479262107837166

[B51] Mahmoodi R. Dadpour M. R. Hassani D. Zeinalabedini M. Vendramin E. Micali S.. (2019). Development of a core collection in Iranian walnut (Ju*glans regia* L.) germplasm using the phenotypic diversity. Sci. Hortic. (Amsterdam) 249, 439–448. doi: 10.1016/j.scienta.2019.02.017

[B52] Marita J. M. Rodriguez J. M. Nienhuis J. (2000). Development of an algorithm identifying maximally diverse core collections. Genet. Resour. Crop Evol. 47, 515–526. doi: 10.1023/A:1008784610962

[B53] McLeod L. Barchi L. Tumino G. Tripodi P. Salinier J. Gros C.. (2023). Multi-environment association study highlights candidate genes for robust agronomic quantitative trait loci in a novel worldwide Capsicum core collection. Plant J. 116, 1508–1528. doi: 10.1111/tpj.16425, PMID: 37602679

[B54] Muñoz-Amatriaín M. Cuesta-Marcos A. Endelman J. B. Comadran J. Bonman J. M. Bockelman H. E.. (2014). The USDA barley core collection: genetic diversity, population structure, and potential for genome-wide association studies. PloS One 9, e94688. doi: 10.1371/journal.pone.0094688, PMID: 24732668 PMC3986206

[B55] Ndjiondjop M. N. Gouda A. C. Eizenga G. C. Warburton M. L. Kpeki S. B. Wambugu P. W.. (2023). Genetic variation and population structure of *Oryza sativa* accessions in the AfricaRice collection and development of the AfricaRice *O. sativa* Core Collection. Crop Sci. 63, 724–739. doi: 10.1002/csc2.20898

[B56] Odong T. Jansen J. Van Eeuwijk F. van Hintum T. J. (2013). Quality of core collections for effective utilisation of genetic resources review, discussion and interpretation. Theor. Appl. Genet. 126, 289–305. doi: 10.1007/s00122-012-1971-y, PMID: 22983567 PMC3555244

[B57] Oksanen J. Kindt R. Legendre P. O’Hara B. Stevens M. H. H. Oksanen M. J.. (2007). The vegan package. Community Ecol. Package 10, 719.

[B58] Omondi E. Barchi L. Gaccione L. Portis E. Toppino L. Tassone M. R.. (2025). Association analyses reveal both anthropic and environmental selective events during eggplant domestication. Plant J. 121, e17229. doi: 10.1111/tpj.17229, PMID: 39918113 PMC11803709

[B59] Pritchard J. K. Stephens M. Donnelly P. (2000). Inference of population structure using multilocus genotype data. Genetics 155, 945–959. doi: 10.1093/genetics/155.2.945, PMID: 10835412 PMC1461096

[B60] Rellstab C. Gugerli F. Eckert A. J. Hancock A. M. Holderegger R. (2015). A practical guide to environmental association analysis in landscape genomics. Mol. Ecol. 24, 4348–4370. doi: 10.1111/mec.13322, PMID: 26184487

[B61] Rellstab C. Keller S. R. (2025). Can we use genomic data to predict maladaptation to environmental change? Mol. Ecol. Res. 25, 1–4. doi: 10.1111/1755-0998.14059, PMID: 39726119

[B62] Rellstab C. Zoller S. Walthert L. Lesur I. Pluess A. R. Graf R.. (2016). Signatures of local adaptation in candidate genes of oaks (*Quercus* spp.) with respect to present and future climatic conditions. Mol. Ecol. 25, 5907–5924. doi: 10.1111/mec.13889, PMID: 27759957

[B63] Risliawati A. Suwarno W. B. Lestari P. Trikoesoemaningtyas Sobir (2023). A strategy to identify representative maize core collections based on kernel properties. Genet. Resour. Crop Evol. 70, 857–868. doi: 10.1007/s10722-022-01469-5

[B64] Roy A. Bandyopadhyay A. Mahapatra A. Ghosh S. Singh N. Bansal K.. (2006). Evaluation of genetic diversity in jute (Corchorus species) using STMS, ISSR and RAPD markers. Plant Breed. 125, 292–297. doi: 10.1111/j.1439-0523.2006.01208.x

[B65] Sah S. K. Reddy K. R. Li J. (2016). Abscisic acid and abiotic stress tolerance in crop plants. Front. Plt. Sci. 7. doi: 10.3389/fpls.2016.00571, PMID: 27200044 PMC4855980

[B66] Saitoh N. Nei M. (1987). The neighbour-joining method: a new method for reconstructing phylogenetic trees. Mol. Biol. Evol. 10, 471–483. doi: 10.1093/oxfordjournals.molbev.a040454, PMID: 3447015

[B67] Sansaloni C. Petroli C. Jaccoud D. Carling J. Detering F. Grattapaglia D.. (2011). Diversity Arrays Technology (DArT) and next-generation sequencing combined: genome-wide, high throughput, highly informative genotyping for molecular breeding of Eucalyptus. BMC Proc. 5, P54. doi: 10.1186/1753-6561-5-S7-P54 22373051

[B68] Sarkar D. Kundu A. Das D. Chakraborty A. Mandal N. A. Satya P.. (2019). Resolving population structure and genetic differentiation associated with RAD-SNP loci under selection in tossa jute (*Corchorus olitorius* L.). Mol. Genet. Genomics 294, 479–492. doi: 10.1007/s00438-018-1526-2, PMID: 30604071

[B69] Sarkar D. Mahato A. K. Satya P. Kundu A. Singh S. Jayaswal P. K.. (2017). The draft genome of *Corchorus olitorius* cv. JRO-524 (Navin). Genomics Data 12, 151–154. doi: 10.1016/j.gdata.2017.05.007, PMID: 28540183 PMC5432662

[B70] Schafleitner R. Lin Y. Dinssa F. F. N’Danikou S. Finkers R. Minja R.. (2022). The World Vegetable Center Amaranthus germplasm collection: Core collection development and evaluation of agronomic and nutritional traits. Crop Sci. 62, 1173–1187. doi: 10.1002/csc2.20715

[B71] Schafleitner R. Lin C.-Y. Lin Y.-P. Wu T.-H. Hung C.-H. Phooi C.-L.. (2021). The world vegetable center okra (*Abelmoschus esculentus*) core collection as a source for flooding stress tolerance traits for breeding. Agriculture 11, 165. doi: 10.3390/agriculture11020165

[B72] Schafleitner R. Nair R. M. Rathore A. Wang Y.-w. Lin C.-y. Chu S.-h.. (2015). The AVRDC–The World Vegetable Center mungbean (*Vigna radiata*) core and mini core collections. BMC Genomics 16, 1–11. doi: 10.1186/s12864-015-1556-7, PMID: 25925106 PMC4422537

[B73] Schmidt T. L. Jasper M. E. Weeks A. R. Hoffmann A. A. (2021). Unbiased population heterozygosity estimates from genome-wide sequence data. Methods Ecol. Evol. 12, 1888–1898. doi: 10.1111/2041-210X.13659

[B74] Schmidt T. L. Thia J. A. Hoffmann A. A. (2024). How can genomics help or hinder wildlife conservation? Annu. Rev. Anim. Biosci. 12, 45–68. doi: 10.1146/annurev-animal-021022-051810, PMID: 37788416

[B75] Shi A. Gepts P. Song Q. Xiong H. Michaels T. E. Chen S. (2021). Genome-wide association study and genomic prediction for soybean cyst nematode resistance in USDA common bean (*Phaseolus vulgaris*) core collection. Front. Plt. Sci. 12. doi: 10.3389/fpls.2021.624156, PMID: 34163495 PMC8215670

[B76] Storey J. Bass A. Dabney A. Robinson D. (2015). Package ‘qvalue’.

[B77] Tajima F. Nei M. (1984). Estimation of evolutionary distance between nucleotide sequences. Mol. Biol. Evol. 1, 269–285. doi: 10.1093/oxfordjournals.molbev.a040317, PMID: 6599968

[B78] Tamura K. Stecher G. Kumar S. (2021). MEGA11: molecular evolutionary genetics analysis version 11. Mol. Biol. Evol. 38, 3022–3027. doi: 10.1093/molbev/msab120, PMID: 33892491 PMC8233496

[B79] Tapia G. González M. Burgos J. Vega M. V. Méndez J. Inostroza L. (2021). Early transcriptional responses in *Solanum peruvianum* and *Solanum lycopersicum* account for different acclimation processes during water scarcity events. Sci. Rep. 11, 15961. doi: 10.1038/s41598-021-95622-2, PMID: 34354211 PMC8342453

[B80] Tchokponhoué D. A. Achigan-Dako E. G. N’Danikou S. Nyadanu D. Kahane R. Houéto J.. (2020). Phenotypic variation, functional traits repeatability and core collection inference in *Synsepalum dulcificum* (Schumach & Thonn.) Daniell reveals the Dahomey Gap as a centre of diversity. Sci. Rep. 10, 19538. doi: 10.1038/s41598-020-76103-4, PMID: 33177634 PMC7658981

[B81] Tchokponhoué D. A. Achigan-Dako E. G. Sognigbé N. D. Nyadanu D. Hale I. Odindo A. O.. (2023). Genome-wide diversity analysis suggests divergence among Upper Guinea and the Dahomey Gap populations of the Sisrè berry (Syn: miracle fruit) plant (S*ynsepalum dulcificum* [Schumach. & Thonn.] Daniell) in West Africa. Plant Genome 16, e20299. doi: 10.1002/tpg2.20299, PMID: 36661287 PMC12807384

[B82] Tefera M. L. Seddaiu G. Carletti A. Awada H. (2025). Rainfall variability and drought in West Africa: challenges and implications for rainfed agriculture. Theor. Appl. Climatol. 156, 1–24. doi: 10.1007/s00704-024-05251-8

[B83] Wang X. Bao K. Reddy U. K. Bai Y. Hammar S. A. Jiao C.. (2018). The USDA cucumber (*Cucumis sativus* L.) collection: genetic diversity, population structure, genome-wide association studies, and core collection development. Hortic. Res. 5, 1–13. doi: 10.1038/s41438-018-0080-8, PMID: 30302260 PMC6165849

[B84] Wang Y. Lü Y. Lü D. Yin L. Wang X. (2024). Climate change and its ecological risks are spatially heterogeneous in high-altitude region: The case of Qinghai-Tibet plateau. CATENA 243, 108140. doi: 10.1016/j.catena.2024.108140

[B85] Wang J. Raskin L. Samuels D. C. Shyr Y. Guo Y. (2015). Genome measures used for quality control are dependent on gene function and ancestry. Bioinformatics 31, 318–323. doi: 10.1093/bioinformatics/btu668, PMID: 25297068 PMC4308666

[B86] Wang Y. Xu Y. Li Q. Wang P. Hu J. Yang L.. (2017). Discovery of related locus on core collection of melon (*Cucumis melo*) fruit character based on GWAS. J. Agric. Biotechnol., 1434–1442.

[B87] Wang R. Zhong Y. Hong W. Luo H. Li D. Zhao L.. (2023). Genetic diversity evaluation and core collection construction of pomegranate (*Punica granatum* L.) using genomic SSR markers. Sci. Hortic. (Amsterdam) 319, 112192. doi: 10.1016/j.scienta.2023.112192

[B88] Wickham H. Chang W. Wickham M. H. (2016). Package ‘ggplot2’. Create elegant Data visualisations using grammar Graphics 2, 1–189.

[B89] Yang Z. Lu R. Dai Z. Yan A. Chen J. Bai Z.. (2018). Analysis of genetic diversity and population structure of a worldwide collection of *Corchorus olitorius* L. germplasm using microsatellite markers. Biotechnol. Biotechnol. Equip. 32, 961–967. doi: 10.1080/13102818.2018.1438852

[B90] Youssef A. F. Younes N. A. Youssef M. (2019). Genetic diversity in *Corchorus olitorius* L. revealed by morphophysiological and molecular analyses. Mol. Biol. Rep. 46, 2933–2940. doi: 10.1007/s11033-019-04754-2, PMID: 30887258

[B91] Zardi G. Nicastro K. Serrão E. Jacinto R. Monteiro C. Pearson G. (2015). Closer to the rear edge: Ecology and genetic diversity down the core-edge gradient of a marine macroalga. Ecosphere 6, 1–25. doi: 10.1890/ES14-00460.1

[B92] Zhang L. Ma X. Zhang X. Xu Y. Ibrahim A. K. Yao J.. (2021). Reference genomes of the two cultivated jute species. Plant Biotechnol. J. 19, 2235–2248. doi: 10.1111/pbi.13652, PMID: 34170619 PMC8541789

[B93] Zia B. Shi A. Olaoye D. Xiong H. Ravelombola W. Gepts P.. (2022). Genome-wide association study and genomic prediction for bacterial wilt resistance in common bean (*Phaseolus vulgaris*) core collection. Front. Genet. 13. doi: 10.3389/fgene.2022.853114, PMID: 35711938 PMC9197503

