# Supplementary material for: Countrywide Corchorus olitorius L. core collection shows an adaptive potential for future climate in Benin
Source: Front Plant Sci. 2025 Sep 30;16:1634672. doi: 10.3389/fpls.2025.1634672 (PMC12518283; doi:10.3389/fpls.2025.1634672)
Supplement: Supplementary file 2 [file DataSheet2.docx]

Supplementary Material

Countrywide *Corchorus olitorius* L. core collection shows an adaptive potential for future climate in Benin

Dèdéou A. Tchokponhoué^1*^, Sognigbé N’Danikou^1,2,3^, Emmanuel Omondi^4^, Spéro Coffi^1^, Belchrist Eliel Sossou^1^, Aristide Carlos Houdegbe^1^, Charlotte A. O. Adje^1^, Nicodeme V. Fassinou Hotegni^1^, M. Eric Schranz^5^, Maarten Van Zonneveld^4^, Enoch G. Achigan-Dako^1*^

^1^Genetics, Biotechnology and Seed Science Unit (GBioS), Laboratory of Plant Production, Physiology and Plant Breeding (PAGEV), School of Plant Sciences, University of Abomey-Calavi, Abomey-Calavi, 01 P.O Box 526, Cotonou, Republic of Benin.

^2^World Vegetable Center, Eastern and Southern Africa, Duluti, Arusha, Tanzania.

^3^Ecole d’Horticulture et d’Aménagement des Espaces Verts, Université Nationale d’Agriculture, Kétou, Bénin.

^4^World Vegetable Center, Headquarters, Shanhua, Tainan City, Taiwan.

^5^Biosystematics Group, Wageningen University and Research, 6708 PB Wageningen, the Netherlands.

# Supplementary Figures


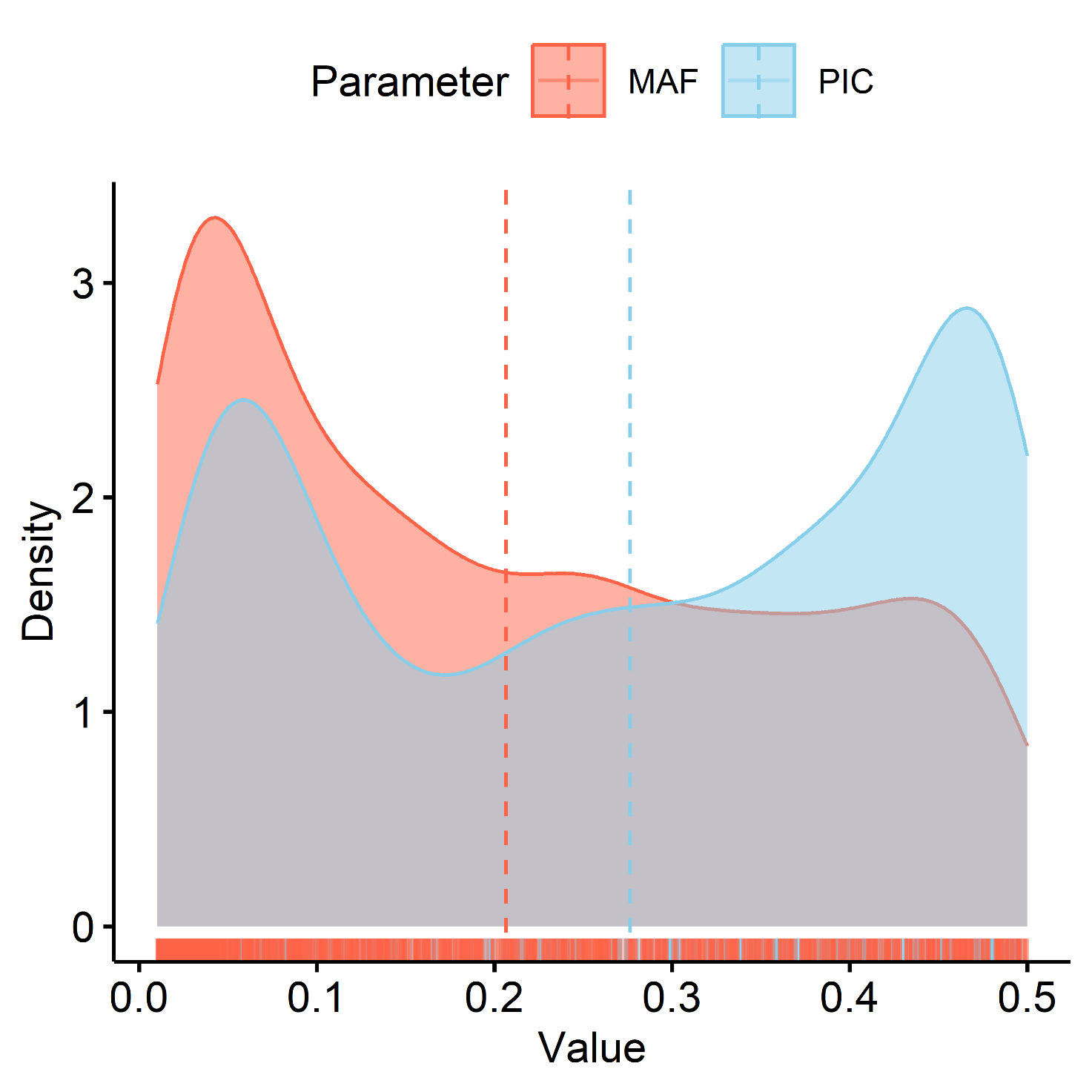


Supplementary Figure **S1 |** Quality attributes parameters (minor allele frequency: MAF and polymorphism information criteria: PIC) of the 1,114 SNP markers used in this study. Pink and blue-colored density curve represented the distribution of minor allele frequency and polymorphism information content, respectively. Colored dashed lines represent the average values.

##
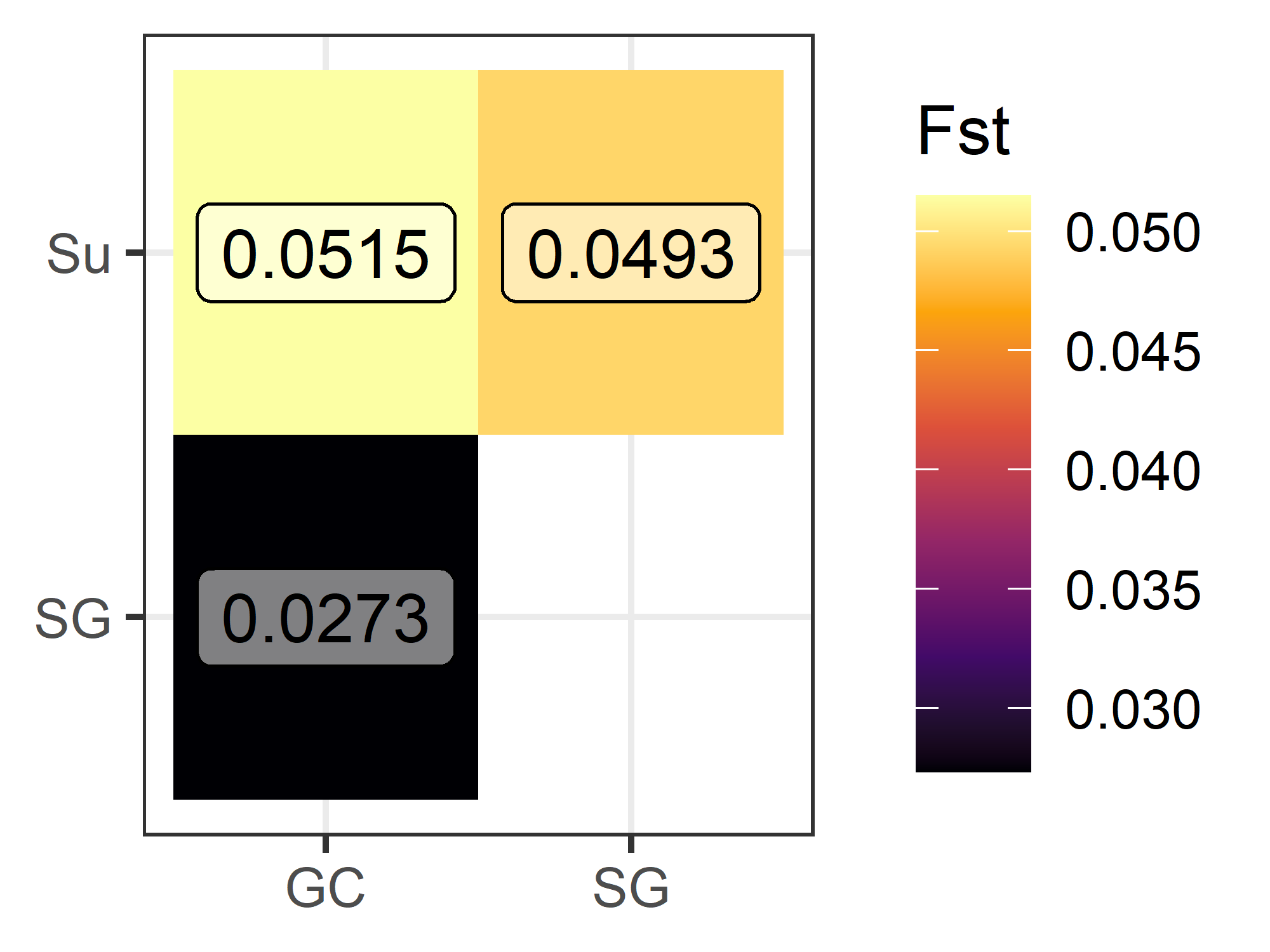


## Supplementary Figure S2. Pairwise-differentiation indices (Fstp) among the studied jute mallow (*Corchorus olitorius*) populations from Benin. GC: Guineo-Congolian population, SG: Sudano-Guinean population and Su: Sudanian population


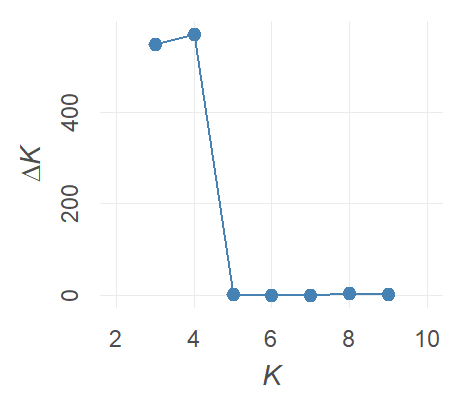


## Supplementary Figure S3. Evanno plot indicating Delta K = mean (|L” (K) / sd (L(K)) from STRUCTURE analyses on 305 accessions of jute mallow (Corchorus olitorius L.) and 1,114 Single nucleotide polymorphism markers.


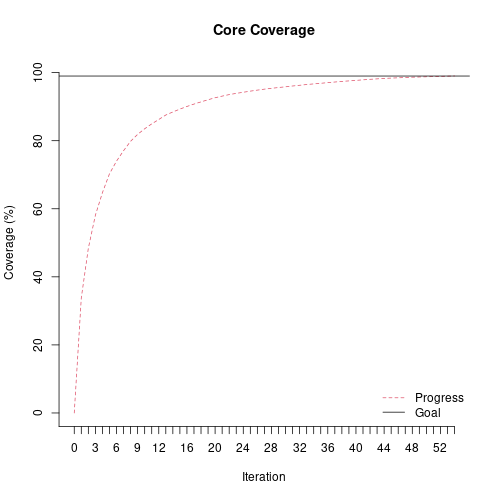


## Supplementary Figure S4. Genotype class coverage accumulation curve in the jute mallow (*Corchorus olitorius*) core set.


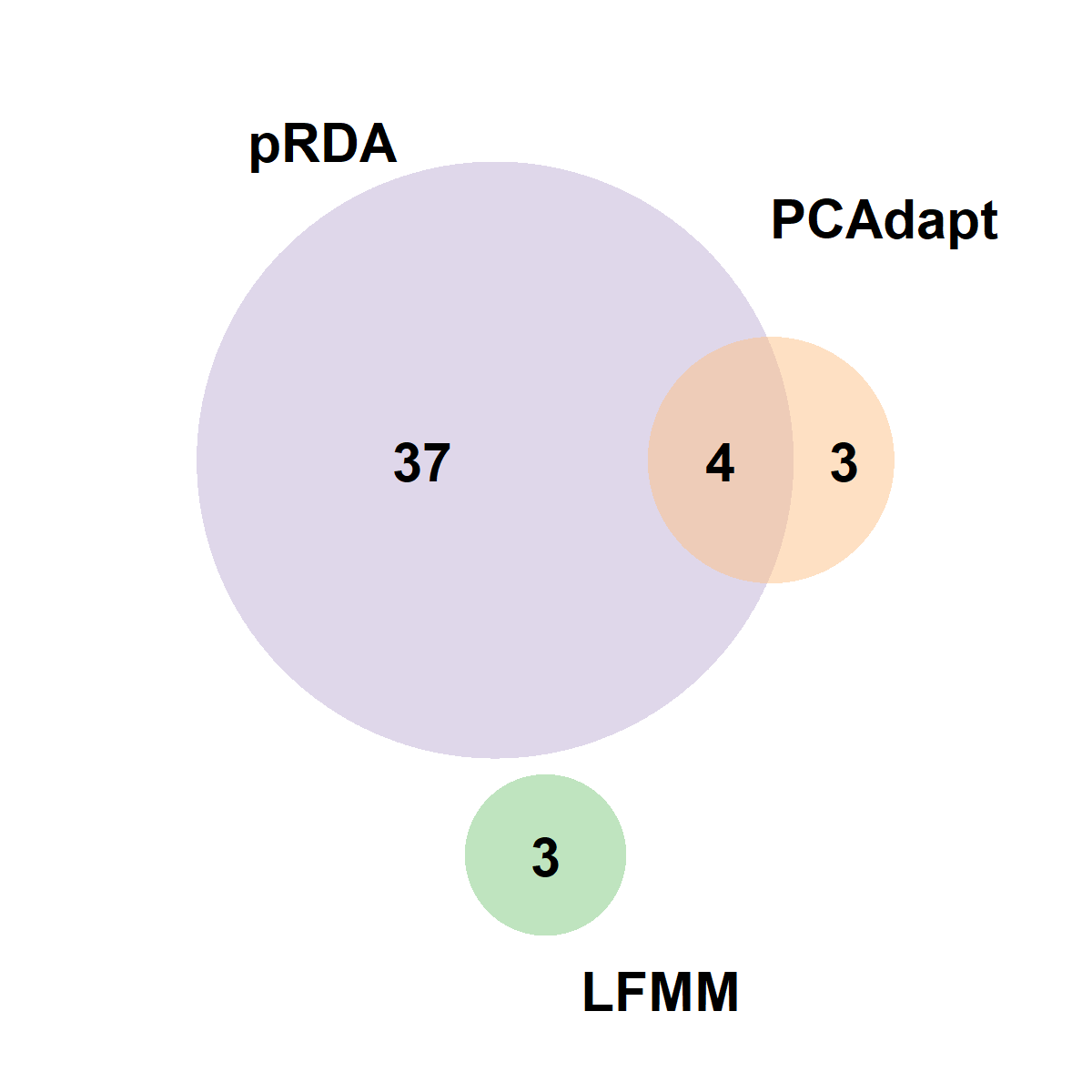


## Supplementary Figure S5. Candidate SNPs detected using three genome scan methods: pRDA - partial redundancy analysis, LFMM - latent factor mixed models and PCAdapt.
